# Large-scale violence in Late Neolithic Western Europe based on expanded skeletal evidence from San Juan ante Portam Latinam

**DOI:** 10.1038/s41598-023-43026-9

**Published:** 2023-11-02

**Authors:** Teresa Fernández-Crespo, Javier Ordoño, Francisco Etxeberria, Lourdes Herrasti, Ángel Armendariz, José I. Vegas, Rick J. Schulting

**Affiliations:** 1 Departamento de Prehistoria, Arqueología, Antropología Social y Ciencias y Técnicas Historiográficas, Universidad de Valladolid, Valladolid, Spain; 2 Research Laboratory for Archaeology and the History of Art, School of Archaeology, University of Oxford, Oxford, UK; 3 Laboratoire Méditerranéen de Préhistoire Europe Afrique – UMR 7269, Centre National de la Recherche Scientifique, Aix-Marseille Université, Maison Méditerranéenne des Sciences de l’Homme, Aix-en-Provence, France; 4 Department of Archaeology and New Technologies, Arkikus, Vitoria-Gasteiz, Spain; 5 Departamento de Medicina Legal y Forense, Universidad del País Vasco (UPV/EHU), Donostia-San Sebastián, Spain; 6 Departamento de Antropología, Sociedad de Ciencias Aranzadi, Donostia-San Sebastián, Spain; 7 Instituto Internacional de Investigaciones Prehistóricas de Cantabria (IIIPC), Universidad de Cantabria, Santander, Spain; 8 Instituto Alavés de Arqueología, Vitoria-Gasteiz, Spain

**Keywords:** Biochemistry, Neuroscience, Endocrinology

## Abstract

This paper explores the nature and extent of conflict in Late Neolithic Europe based on expanded skeletal evidence for violence from the San Juan ante Portam Latinam rockshelter in present-day Spain (ca. 3380–3000 cal. BC). The systematic osteological re-examination has identified 65 unhealed and 89 healed traumas—of which 77 were previously undocumented—consistent with aggression. They affect 23.1% of the 338 individuals represented. Adolescent and adult males are particularly affected (44.9% of the 107 identified), comprising 97.6% of unhealed trauma and 81.7% of healed trauma recorded in individuals whose sex could be estimated and showing higher frequencies of injuries per individual than other demographic subgroups. Results suggest that many individuals, essentially men, were exposed to violence and eventually killed in battle and raids, since warriorship is mainly restricted to this demographic in many societies. The proportion of casualties is likely to have been far greater than indicated by the 10.1% individuals exhibiting unhealed trauma, given the presence of isolated cases of unhealed postcranial trauma and of arrowheads potentially having impacted into soft tissues. This, together with skeletal indicators of poor health and the possible socioeconomic outcomes evidenced in the region, suggest wider social impacts, which may relate to a more sophisticated and formalized way of warfare than previously appreciated in the European Neolithic record.

## Introduction

Small- and medium-scale collective violence, in the form of confrontations between neighboring groups, may be as old as humankind [e.g.,^1, 2^]. By contrast, larger-scale organized and sanctioned lethal violence between sociopolitical groups (i.e., warfare), seems to be associated with certain socio-economic conditions that generally accompanied the shift to a farming economy,^3^ such as higher population densities and greater degrees of sedentism^4, 5^, in parallel to the increasing importance of resource ownership and property that led to the concentration of resources and power [e.g.,^6, 7^].

Nevertheless, the nature and the extent of European Neolithic warfare remains poorly understood. Unfortunately, many forms of behavior associated with conflict fail to generate material evidence or leave traces that are easily recognizable, especially when settlements are virtually absent in the local record. Despite the increasing evidence of massacres and other mass burials, such sites remain relatively rare [e.g.,^8, 9^]. They usually include no more than two or, very occasionally, up to 30–40 individuals with unhealed trauma, suggesting small- or medium-scale violent events [e.g.,^10, 11^]. In some contexts (e.g., Linearbandkeramik (LBK) in central Europe, Bruebach-Oberbergen, western Bischheim and Early Michelsberg in the Rhine valley, or the Late Neolithic in the Ebro valley), the relative frequency and broad contemporaneity of such findings has served as proof of widespread short-term unrest, traditionally linked to environmental crises and/or population pressure [e.g.,^12–15^]. However, it is unknown whether the rationale behind such violence responds to a common regional underlying cause or to particular triggers in each case [e.g.,^16, 17^].

The Late Neolithic funerary rockshelter of San Juan ante Portam Latinam (henceforth SJAPL) in north-central Iberia is one of the most promising sites to represent the largest violence-related burial event known for the European Neolithic. Nevertheless, the scale and nature of violence at the site remains disputed. The site was originally interpreted as a massacre (i.e., the indiscriminate killing of helpless or unresisting people)^18^ based on the haphazard and interwoven deposition of many bodies in a single mass with minimal soil separating them, combined with the original identification of five unhealed arrowhead injuries, at least 41 potential cases of arrowheads having impacted bodies and a single case of unhealed cranial trauma [i.e.,^19–22^]. However, such evidence appeared to be too limited to support a common cause of death for the estimated minimum number of 338 individuals present^23^. New studies have recently challenged previous interpretations, suggesting that the assemblage’s demography, characterized by very high rates of adolescent and adult males, may better fit with one or more violent engagements, in combination with some attritional mortality^22^. This idea would be in line with the emphasis on violence at a distance apparently suggested by the projectile injuries, which is unusual in other European Neolithic massacres, wherein cranial injuries feature strongly^10, 24^. Healed trauma recorded in SJAPL, originally including 53 healed cranial traumas, eight healed arrowhead injuries and six healed parry fractures^19^, may conversely support the important role of close-contact encounters in a context of recurring conflict of less lethal intent^22^.

It is not uncommon for unusual or atypical burials to be subsumed under the umbrella of massacres, even if they are likely to have been the result of very different processes, violence-related or not^3^. Unfortunately, the identification of unhealed trauma is not always straightforward, especially when dealing with large, fragmented and mixed human skeletal assemblages. Even with small and well-preserved sites, the identification of lethal violence often relies on the archaeological context. For example, in the Early Neolithic LBK, where most individuals were interred as single inhumations or cremation, multiple burials may be suggestive of violence-related events^16^. In such cases, despite the fact that unhealed injuries rarely affect more than half of the individuals deposited, a common cause of death is generally assumed on the not-unreasonable basis that skeletal signs of violence only ever reflect the minimum number of injuries present originally^3^.

However, the Late Neolithic presents a far more complex scenario. The gathering of multiple individuals in a shared burial place (i.e., megalithic graves, caves and rock-shelters, etc.) became the dominant funerary treatment across much of the continent during this period [e.g.,^25^]. In such a context, casualties of violent encounters included in such cumulative burial sites may be easily effaced by attritional features (i.e., extensive commingling, disarticulation and fragmentation). Even cases involving a catastrophic violence-related event can be misunderstood as a normative cumulative burial site, especially if bodies remained unburied for a time and some degree of disarticulation and fragmentation exists, or if skeletal evidence for violence is not dominated by blunt force trauma. Associated arrowheads, for example, may be interpreted as grave offerings.

Given the challenging nature of the archaeological record at SJAPL, a systematic reassessment of the skeletal assemblage has been conducted in order to define the extent and impact of violence at the site and explore its broader implications for our understanding of Neolithic warfare. Of particular importance is the re-examination of crania for evidence of trauma, given its surprisingly low incidence relative to the number of projectile injuries, making it unusual in a European Neolithic context.

## The site

**Figure 1:**
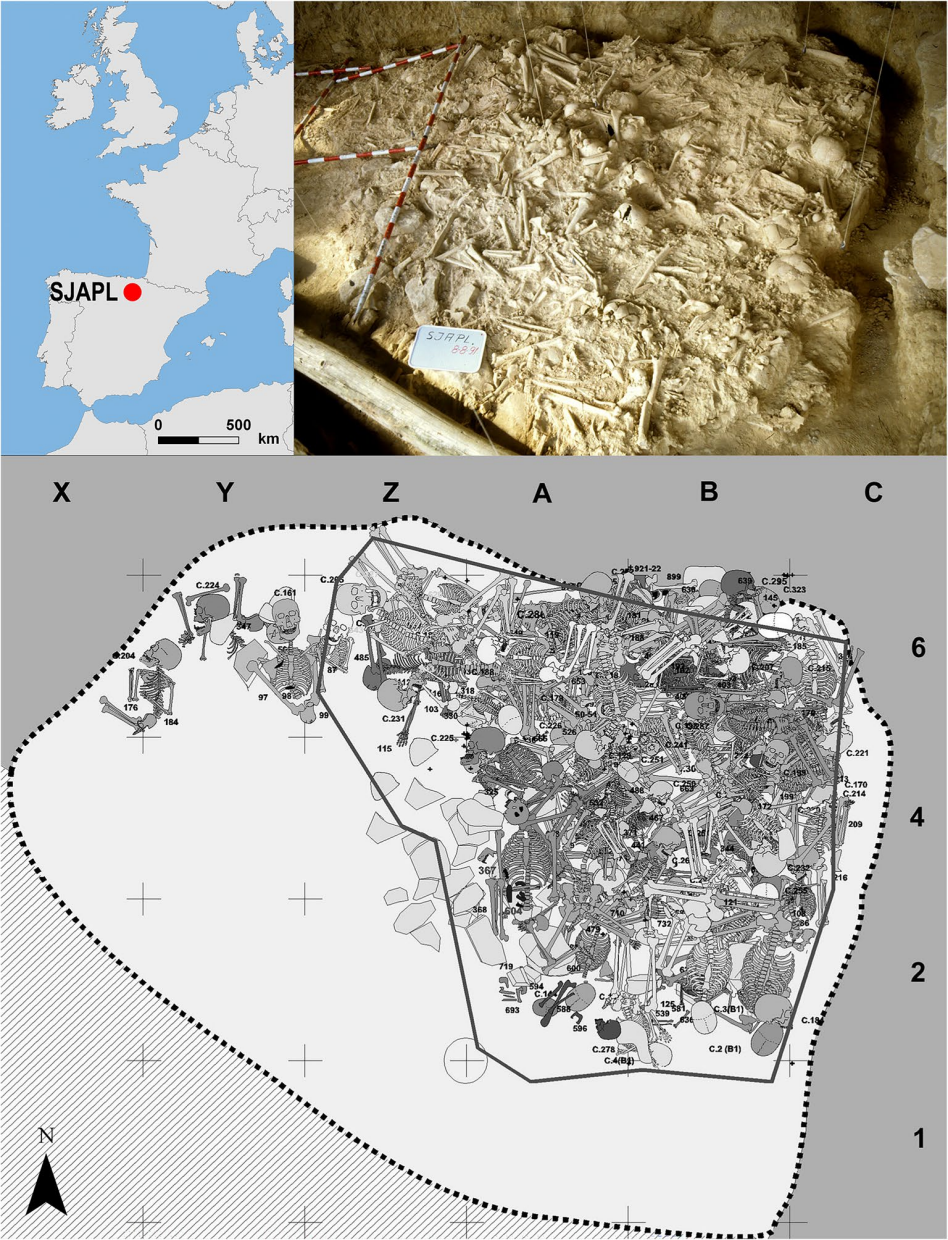
Top left Location of SJAPL in north-central Iberia. Top right Surface view of the eastern corner of the burial deposit before being excavated. Bottom Plan where the best-preserved skeletons are reconstructed.

The Late Neolithic funerary rockshelter of SJAPL is located in the Rioja Alavesa region of north-central Iberia (SW Europe) (Fig. 1). It was discovered in 1985 when a bulldozer was widening a track and accidentally uncovered human remains. Rescue excavations were carried out that year and in 1990 and 1991, after the controlled explosion of the collapsed roof (a 25-ton sandstone block) that sealed the site. They revealed a ca. 20 m^2^ rockshelter with a huge accumulation of human remains, including 90 complete and 31 incomplete skeletons, 255 articulated skeletal segments and thousands of apparently isolated bones^26^.

A minimum of 338 individuals (201 nonadults and 137 adults, based on crania), with a clear predominance of males (107 out of the 153 adolescents and adults whose sex could be estimated, i.e., 70%)^19^ were buried randomly interwoven and occasionally in atypical positions (including prone and unnaturally flexed positions) (cf. Fig. 1). They were found together with 52 flint arrowheads, 64 blades, two polished stone axes, three pebbles, five bone awls and some personal ornaments^23^. These materials attribute the deposit to the Late Neolithic, confirmed by 16 radiocarbon dates on human bone, which a Bayesian model places as lying between 3380 and 3000 cal. BC^22^. At that time, the region witnessed the emergence of funerary variability, mainly consisting of megalithic graves and funerary rockshelters, and which seem to have marked a socially meaningful division^27^.

The site has been understood as a primary burial because the decomposition of the bodies (with the exception of the uppermost individuals) mostly occurred on a filled space, which was potentially created by the superimposition of bodies (with as many as eight layers in some areas) within the grave and not by soil or other elements like shrouds^19^. Severe interweaving and extensive fragmentation of bones (largely resulting from the collapsed roof) presented serious handicaps for the identification of further skeletal connections. Additional difficulties were presented by the extensive damage and skeletal loss in the peripheral areas caused by the bulldozer that accidentally uncovered the deposit^19^.

However, some irregularities in bone representation might point to the existence of a number of non-primary burials. In this regard, a predominance of skulls—which were used to estimate the minimum number of individuals – followed by long bones especially femora – and a clear underrepresentation of pelvic bones, ribs, vertebrae and small bones of the hands and feet have been reported^19, 28^. So far, these anomalies have been interpreted as a result of either the removal of bones from the tomb or of inherent differences in mechanical resistance. Nevertheless, it is possible that some individuals at SJAPL might have remained unburied for a time. However, the lack of traces of carnivore activity, apart from very limited rodent gnawing, suggests that individuals were not exposed for long [e.g.,^17^]. This is further supported by the abundance of anatomical connections, including labile articulations. There is no clear evidence that the surplus of skulls reflects either trophy taking or ancestor veneration practices. While a possible ‘nest’ of skulls has been described at the site, it is unclear whether this is intentional or a result of postdepositional displacements of skulls.

The site has been considered exceptional in a European context for the number of individuals, mainly adolescent and adult males, originally reported to exhibit skeletal signs of violence (i.e., eight healed and five unhealed cases of arrowhead injuries, with an additional two possible cases, together with 53 healed and one unhealed cranial traumas, and six healed parry fractures^19^). Unhealed trauma, together with the aforementioned unusual disposition of skeletons, was originally proposed as evidence of a potential common cause of death, i.e., a massacre^18^. However, available skeletal evidence for unhealed trauma seems to be low and the demographic profile of the site does not fit well with that expected for a massacre, which tends to replicate a natural population profile (as seen in other Neolithic violence-related mass graves [e.g.,^10^]).

In this regard, a study based on agent-based demographic modeling recently suggested that the SJAPL assemblage may be the result of one or more catastrophic events, particularly battles and/or raids, in combination with some attritional mortality^22^. This study considered that, despite the apparently low frequency of originally published unhealed trauma, the number of victims could have been much larger based on the identification of striations and fractures consistent with impacts in 36 out of 42 of the 52 isolated arrowheads recovered at the site (the remaining 10 were not subjected to use-wear analysis^20, 21^) and on the observation of cases of close spatial association between 24 of these projectiles and skeletal elements^19^. This would make a total of at least 41 potential cases of arrowheads having impacted bodies and, presumably, having entered the rockshelter either embedded in bone or in soft tissues (Table S1). This is a minimum estimate, since presumably other arrows would have been removed prior to burial. Unfortunately, the fact that available radiocarbon dates fall within the late fourth millennium BC plateau in the calibration curve, prevented Bayesian modeling conducted in the same study from attaining the required precision to distinguish between a single event or a short period of time (months or years) on the one hand, or deposition over one to two centuries between ca. 3380–3000 cal. BC on the other^22^.

## Results

### Cranial trauma

A total of 107 cranial injuries have been identified, of which 48 (44.9%) are unhealed and 59 (55.1%) healed (Tables 1 and S2). Of these, 43—one unhealed and 42 healed—had previously been recorded [^19, 23^: appendix] together with another 11 cases which either were apparently missing in the collection (n = 2), have not been observed on bone (n = 6) or have been interpreted not to be an injury (n = 3) during the re-examination (and, consequently, are not considered, described nor included in tables in this paper).

**Table 1:** Cranial injuries identified in SJAPL in this study, classified by injury type and whether healed or unhealed.

		Injury type^a^				Total
		Blunt-force	Sharp-force	Penetrative	Trepanation	
Healing	No	45	2	–	1	48
	Yes	51	3	2	3	59
Total		96	5	2	4	107

^a^Classified following Smith.^29^

**Figure 2:**
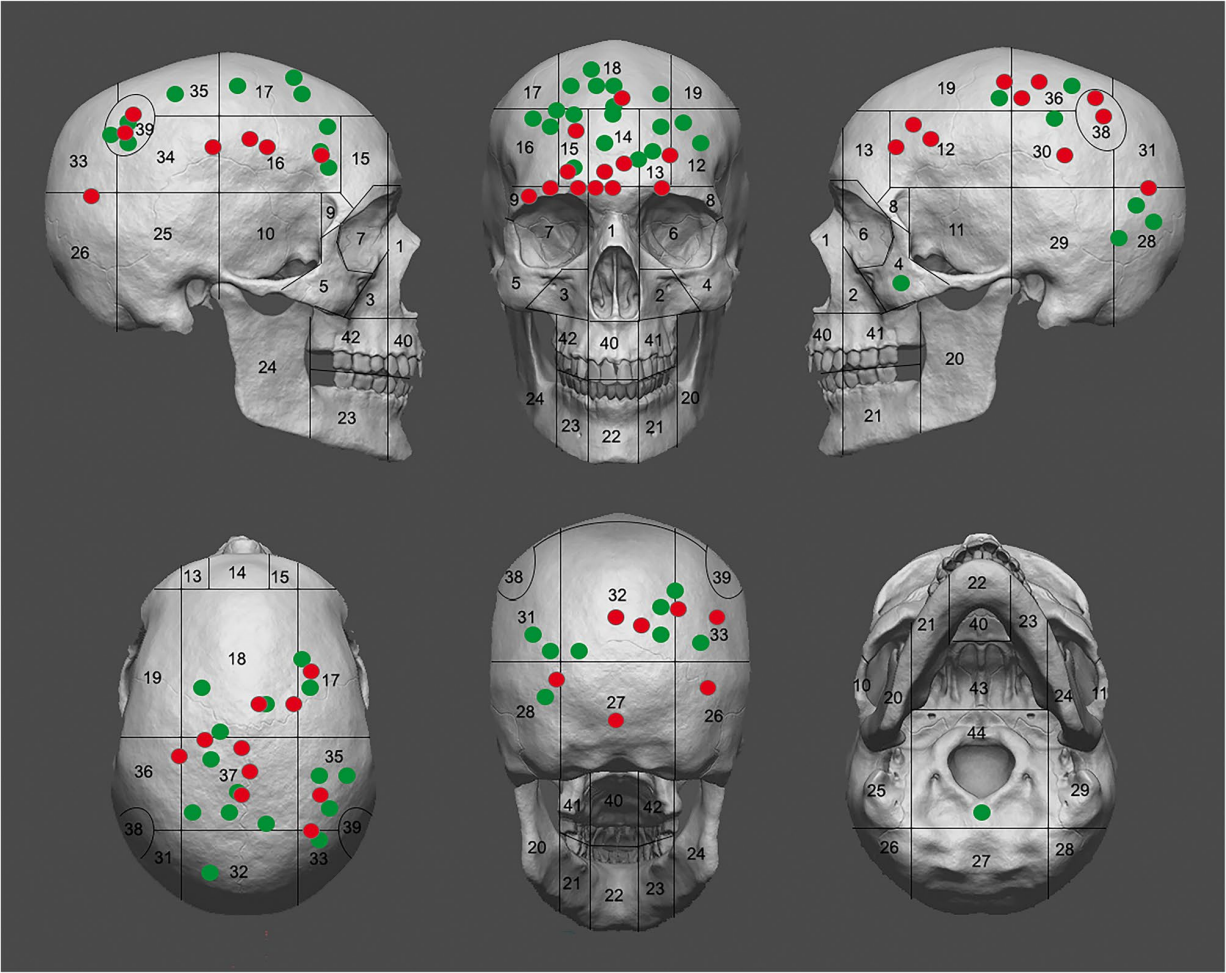
Locational distribution of unhealed (red dots) and healed (green dots) cranial injuries in SJAPL documented in this study (including a number of cases previously described or recorded [^19, 23^: appendix]), with reference to the zonation method proposed by Hussain et al.^32^ to record injuries to the skull. Injury locations are displayed once to avoid duplication, regardless their potential observation in different views. Skull 3D model images created by and courtesy of S. Abad.

**Figure 3:**
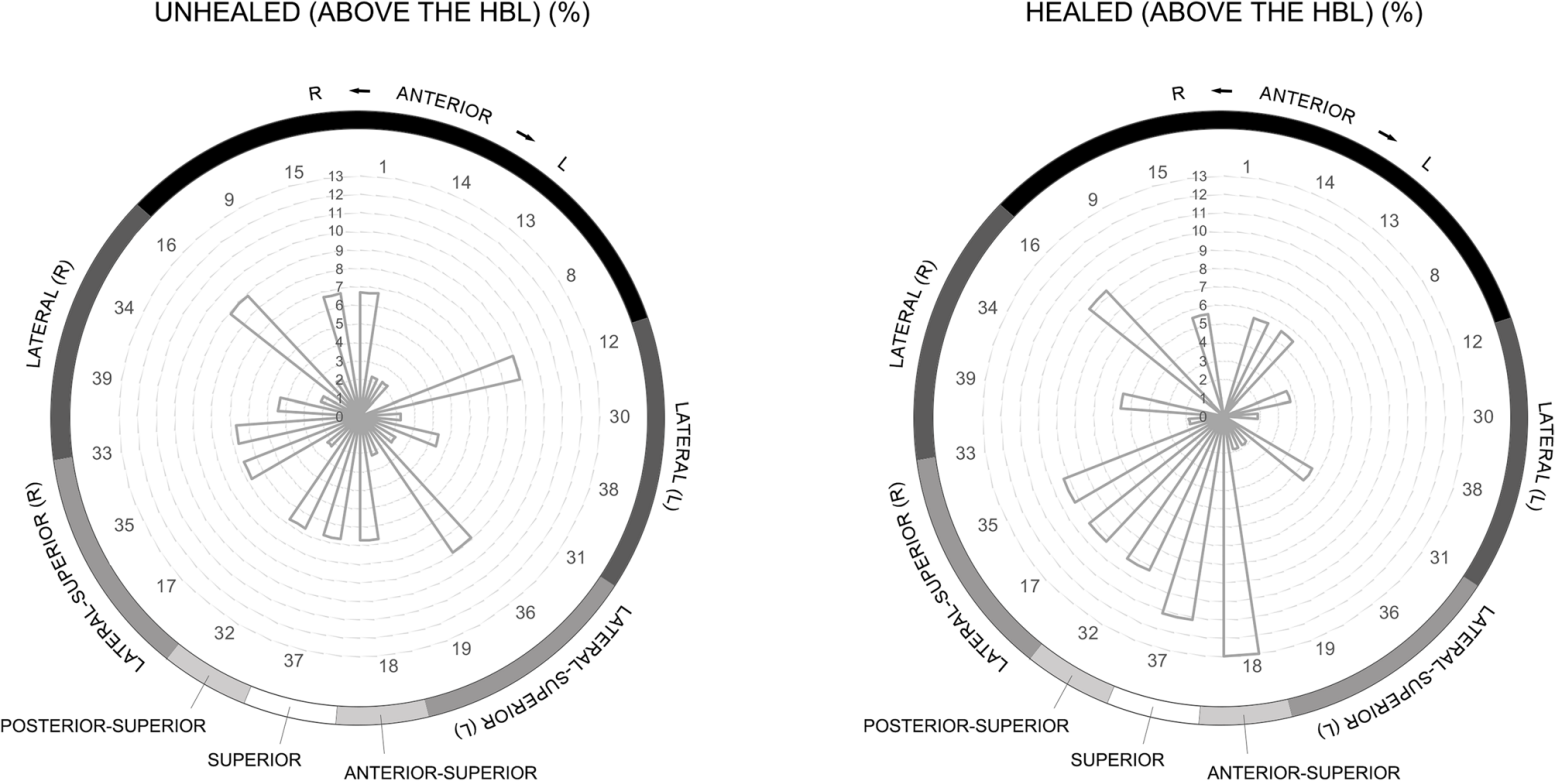
Wind rose diagram showing frequencies (%) of unhealed (left) and healed (right) cranial injuries documented above the hat brim line (HBL) in SJAPL, distributed according to Hussain et al.’s zonation method^32^.

Ninety-eight cases (91.6%) are located above the hat brim line (HBL) (i.e., top of the cranium including the frontal, upper parietals and upper portion of the occipital), where wounds have traditionally been assumed to be more likely due to deliberate blows^30^, despite recent research suggesting that the HBL rule should be used with caution^31^. The exceptions are three unhealed and six healed traumas, including an arrowhead injury affecting the occipital bone [see^23^]. Frontal and parietal bones are affected preferentially (n = 101, 94.4%), while temporo-occipital and maxillofacial regions seem to be rarely implicated (n = 6, 5.6%, of which three represent unhealed cases, all found on the occipital bone). However, the preservation rates of the maxillofacial region are substantially lower, so injuries to these regions are probably under-represented. Locational distribution of unhealed trauma clearly shows predominance of lateral and anterior aspects, whereas healed trauma more often affects superior and upper anterior aspects (Figs. 2 and 3). Lateral left and right cranial aspects are similarly affected by both unhealed (n = 10 vs. 8) and healed (n = 7 vs. 11) trauma. The anterior cranium is more affected than the posterior for both unhealed (n = 12 vs. 6) and healed (n = 16 vs. 9) trauma, on a total of 196 largely complete frontals and 193 occipitals. The difference in location is statistically significant when healed and unhealed injuries are combined (*χ*^*2*^ = 4.2, df = 1, *p* = 0.041, *V* = 0.104), though this does not take into account the larger size of the frontal compared to the occipital, hence presenting a larger target area.

**Figure 4:**
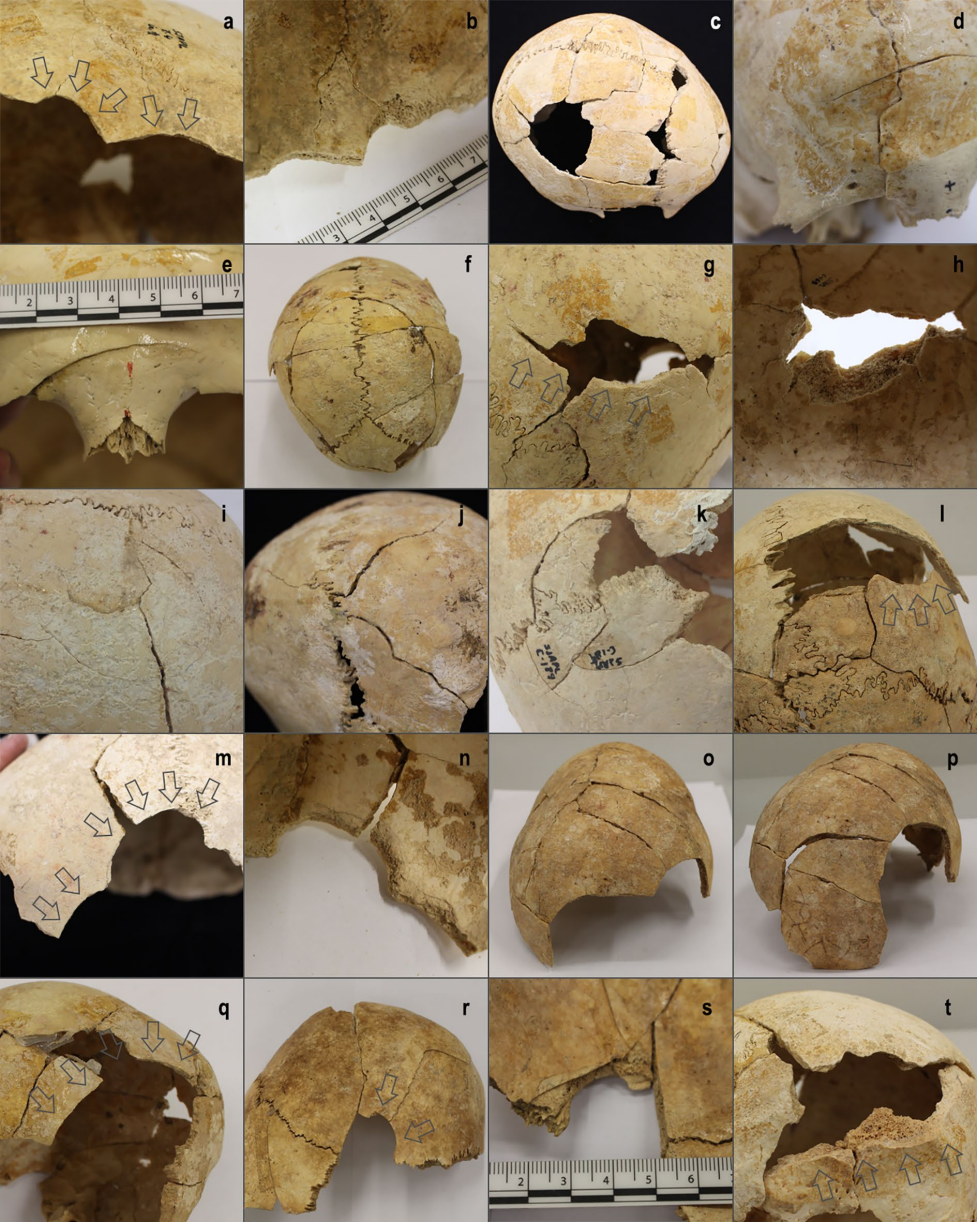
Examples of unpublished unhealed cranial injuries documented in SJAPL in this study. (**a**–**b**) Blunt-force traumas in the right parietal bone of cranium C001(Z4). (**c**) Large blunt-force trauma affecting the right parietal and frontal bones of cranium C002. (**d**) Linear fractures in the right half of the frontal bone of cranium C003(B1). (**e**) Linear fracture above the nasion of cranium C130. (**f**) Large blunt-force trauma affecting both left and right parietal and the occipital bones of cranium C148. (**g**–**h**) Sharp-force trauma in the right parietal bone of cranium C168. (**i**) Blunt-force trauma in the left parietal bone of cranium C169. (**j**) Radiating linear fractures in the occipital bone of cranium C172. (**k**) Blunt-force trauma affecting the right parietal and occipital bones of cranium C189. (**l**) Blunt-force trauma in the right parietal bone and linear fractures affecting the occipital bone of cranium C198. (**m**–**n**) Blunt-force traumas in the left half of the frontal bone of cranium C199. (**o**–**p**) Large blunt-force traumas affecting both the right parietal and occipital bones of cranium C225. (**q**) Blunt-force trauma in the left parietal bone of cranium C229. (**r**–**s**) Blunt-force trauma and linear fractures affecting both the frontal and left parietal bones of cranium C326. (**t**) Sharp-force trauma in the occipital bone of cranium C334. Panels (**a**), (**c**–**g**), (**i**–**m**), (**o**–**r**), (**t**) in ectocranial view; panels (**b**), (**h**), (**n**), (**s**) in endocranial view.

Most lesions (n = 96) correspond to depressed and/or linear fractures attributable to blunt-force trauma (e.g., caused by blunt stone maces, axes and adzes; wood, bone or antler clubs; sling-shot or thrown stones, etc.). In addition, there are 11 cases interpreted as two unhealed and three healed sharp-force traumas, two healed penetrating injuries caused by arrowhead impacts, and one unhealed and three healed trepanations (Tables 1 and S2). Unhealed blunt-force traumas mainly displayed medium size oval or curved-shaped depressions (average max. diameter, including incomplete examples: ≥ 3.6 ± 2.0 cm, ranging from 1.5 to 12.1 cm, n = 30) with internal beveling (i.e., complete fractures) and/or radiating linear fractures with oblique angles and smooth and/or beveled edges (Fig. 4). Comminuted bone fragments are also occasionally observed. Healed blunt-force traumas are generally represented by small circular and oval depressions (average max. diameter, including incomplete examples: ≥ 1.4 ± 1.0 cm, ranging from 0.2 to ≥ 6 cm, n = 58) with rare involvement of the inner table (i.e., incomplete fractures).

Cranial injuries affect 71 of the 338 individuals at SJAPL (21.0%). Of these, 13 show multiple unhealed traumas, nine show multiple healed traumas and three show the co-occurrence of both unhealed and healed injuries (Table S2). As a result of cranial fragmentation and the locational distribution of injuries, Puppe’s rule to determine the temporal sequence in which those were inflicted^33^ could be applied in only seven cases in which two unhealed blunt-force traumas showed a clear sequence. In these, first blows predominantly affected lateral and lateral-anterior aspects (6 out of 7), whereas second blows mainly affected lateral-posterior and posterior aspects (5 out of 7).

The individuals affected by cranial trauma comprise 42 adolescents (12–19 years of age) and adults (≥ 20 years of age) identified as male/probable male, nine adolescents and adults identified as female/probable female, and nine adolescents and adults, four children (7–11 years of age) and seven infants (0–6 years of age) of unknown sex. Unhealed cranial injuries are present in 19 adolescent and adult males, one adult female, and four adolescents/adults, three children and four infants of indeterminate sex (n = 31), whereas healed cranial injuries are present in 26 adolescent and adult males, eight adolescent and adult females, and five adolescents/adults, one child and three infants of indeterminate sex (n = 43).

### Postcranial trauma

A total of 47 postcranial injuries have been identified, of which 17 are unhealed and 30 healed (Tables S3 and S4). Of these, 34—five unhealed and 29 healed—had previously been described or recorded [^19, 23^: appendix] together with another three cases which have not been found in the collection during the re-examination (and, therefore, are not considered, described nor included in the analysis). Injuries preferentially affect long bones of the upper and lower limbs (n = 22, 46.8%). However, there are also a number of cases affecting clavicles (n = 3), scapulae (n = 2), vertebrae (n = 3), ribs (n = 6), coxal bones (n = 5), and both hand (n = 2) and foot (n = 4) bones. Lower rates of traumas affecting long bone metaphyses and epiphyses (n = 3) and other generally poorly-preserved skeletal elements, such as scapular bodies, ribs and vertebral arches, are probably due to the fact that these regions are more compromised by postmortem and recent damage.

**Figure 5:**
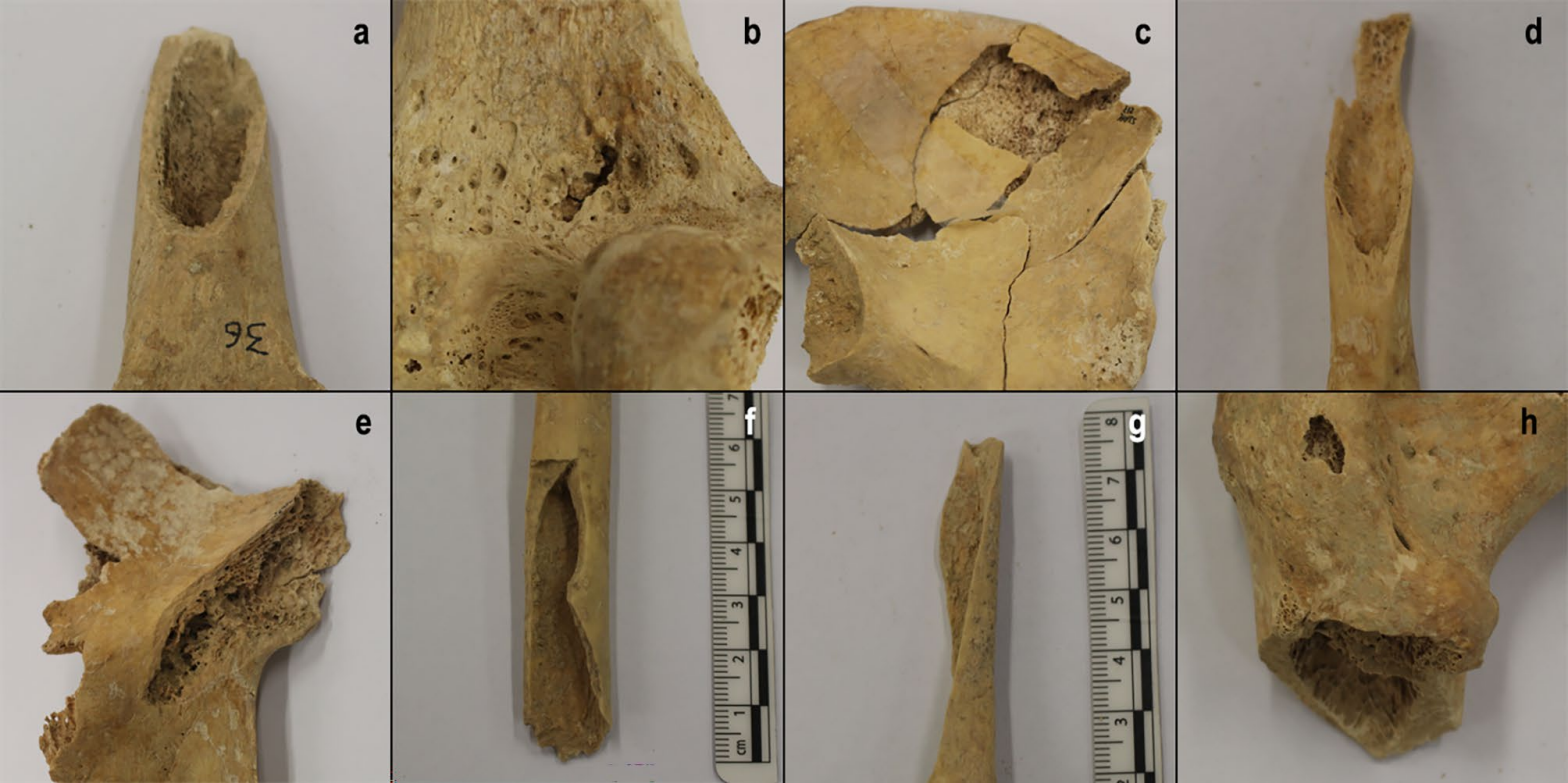
Examples of unpublished unhealed postcranial injuries documented in SJAPL in this study. (**a**) Blunt-force trauma in the diaphysis of left femur SJAPL.36(b). (**b**) Penetrative injury on the distal metaphysis of left femur SJAPL.161. (c) Linear fractures in the left coxal bone SJAPL.217. (**d**) Blunt-force trauma in the diaphysis of the right 3rd metacarpal bone SJAPL.377. (**e**) Blunt-force trauma in the right coxal bone SJAPL.408. (f) Blunt-force trauma in the right radius SJAPL.A1.35. (**g**) Blunt-force trauma in the distal metaphysis of the right radius SJAPL.B1(b). (**h**) Blunt-force trauma in the diaphysis of the left femur SJAPL.SIN_SIGLA(e).

Most lesions (n = 31, 66.0%) consist of either spiral- and V-shaped fractures with oblique angles and smooth and/or beveled edges when unhealed (n = 9) (Fig. 5), or consolidated fractures with visible calluses when healed (n = 22). Among the latter are four ulna fractures tentatively identified as parry fractures (in the absence of the ipsilateral radius), one radius fracture identified as a Galeazzi fracture and one paired rotational fracture affecting both the ulna and the radius (which were both identified as parry fractures in previous studies). They can be interpreted as the result of a direct impact to the forearm, usually when this is raised to protect the face, although a differential diagnosis (e.g., fracture caused by an indirect force, stress fractures) should be considered particularly in the last two cases^34^. The remaining sixteen cases are interpreted as two unhealed sharp-force traumas, one shoulder dislocation, and six unhealed and seven healed penetrative injuries. Among the latter, at least 11 are clearly caused by an arrowhead impact^19, 23^, which add to the aforementioned two cases on crania. Concerning the possible trajectories of arrowheads, these are consistent with impacts from behind in most cases (69.2%, n = 9), the remaining being from the front (n = 2), from the right (n = 1) and unknown (n = 1).

All postcranial traumas identified affect adolescent or adult (i.e., skeletally mature) individuals. Recording of healed injuries to juveniles may be partially obscured due to rapid bone turnover rates^35^ and the greater fragility of immature bones (which would affect both healed and unhealed trauma)^36^, although it is also possible that infants and children were much less exposed to physical violence^37^, though note that 11 infants/children exhibit cranial trauma, seven of which showed no signs of healing.

Sex determination is constrained by the fact that postcranial traumas are mostly represented by incomplete, isolated bones. However, there are 17 cases where affected elements can be attributed to complete or partially complete skeletons. These correspond to 11 males (of which five show only unhealed, four only healed and two the co-occurrence of unhealed and healed injuries) and three females (all showing healed injuries only) (Table S4).

### Combined data

Cranial traumas are clearly more abundant than postcranial traumas in SJAPL, showing similar proportions in both unhealed (73.8 vs. 26.2%) and healed (66.3 vs. 33.7%) injuries.

Combining both cranial and postcranial data results in at least 78 out of the 338 individuals represented at SJAPL (i.e., 23.1%) being affected by violence-related trauma. This prevalence is similar to that obtained by including cranial trauma only (21.0%) since most postcranial injuries cannot be linked to a specific individual/cranium. The affected individuals are predominantly identified as adolescent and adult male/probable male, comprising 48 of the 107 cases documented in SJAPL (i.e., 44.9%). However, trauma also affects 11 of the 46 adolescents and adults identified as female/probable female (23.9%) and 11 of the 131 infants and children of unknown sex documented at the site (8.4%). Unhealed injuries are present in 22 adolescent and adult males, one adult female, and four adolescents/adults and seven children/infants of indeterminate sex (n = 34), whereas healed injuries are present in 30 adolescent and adult males, 10 adolescent and adult females, and five adolescents/adults and four children/infants of indeterminate sex (n = 49). Differences in the rates of trauma between sexes are statistically significant for the combined data (*χ*^2^ = 6.0, df = 1, *p* = 0.015, *V* = 0.197) and for unhealed injuries (*χ*^2^ = 7.1, df = 1, *p* = 0.008, *V* = 0.236), but not for healed injuries (*χ*^2^ = 0.7, df = 1, *p* = 0.416, *V* = 0.066).

Considering the number of injuries, adolescent and adult males exhibit 40 unhealed trauma and 49 healed trauma. These represent 74.1% of unhealed trauma and 70.0% of healed trauma recorded in individuals (i.e., 54 and 70 traumas for 34 and 49 individuals, respectively), and 97.6% of unhealed trauma and 81.7% of healed trauma recorded in those whose sex could be estimated (i.e., 41 and 60 traumas for 23 and 40 individuals, respectively). This predominance is consequently reflected in frequencies of injuries per individual, where adolescent and adult males show notably higher frequencies of both unhealed (1.8) and healed (1.6) injuries compared to females and children/infants (ca. 1.1 in most categories). It is worth noting that, among males, unhealed injuries are more commonly found among adolescents (which also show the highest frequency of injuries per individual, 2.2) and healed injuries among young and middle adults (Table 2).

**Table 2:** Sex and age distribution of individuals showing unhealed and healed injuries in SJAPL (this study).

		Unhealed					Healed				
		Sex			Subtotal (by age)	Frequency of injuries per individual (by age)	Sex			Subtotal (by age)	Frequency of injuries per individual (by age)
		Male	Female	Indet			Male	Female	Indet		
Age category (years)	Infant (0–6)	–	–	4 (4)	4 (4)	1.0	–	–	3 (3)	3(3)	1.0
	Child (7–11)	–	–	3 (4)	3 (4)	1.3	–	–	1 (2)	1(2)	2.0
	Adolescent (12–19)	8 (19)^a^	–	1 (1)	9 (20^a^)	2.2	6 (9)^a^	5 (6)	1 (1)	11(15^a^)	1.4
	Younger adult (20–39)	9 (12)^b^	1 (1)	–	10 (13^b^)	1.3	10 (17)^b^	2 (2)	–	12 (19^b^)	1.6
	Middle adult (40–59)	5 (9)^a^	–	1 (2)	6 (11^a^)	1.8	14 (23)^a^	2 (2)	–	16 (25^a^)	1.6
	Old adult (> 60)	–	–	–	*–*	*–*	–	1 (1)	–	1 (1)	1.0
	Indeterminate adult (> 20)	–	–	2 (2)	2 (2)	1.0	–	–	4 (4)	4 (4)	1.0
Subtotal (by sex)		22(40^c^)	1 (1)	11 (13)			30 (49^*c*^)	10 (11)	9 (10)		
Frequency of injuries per individual (by sex)		1.8	1.0	1.2			1.6	1.1	1.1		

^a^Includes two individuals with co-occurrence of both unhealed and healed injuries.

^b^Includes one individual with co-occurrence of both unhealed and healed injuries.

^c^Includes four individuals with co-occurrence of both unhealed and healed injuries.

The total number of injuries is shown in brackets.

A combined total of 27 of 338 individuals (8.0%) show multiple trauma, including 23 adolescent and adult males, one adolescent female and one adult and two children of indeterminate sex. Of these, 12 show multiple unhealed injuries (10 males and one adult and one child of indeterminate sex), 10 multiple healed injuries (eight males, one female and a child of indeterminate sex) and five the co-occurrence of both (all males). Seven individuals (six males—C198, C212, C218, C221, C225 and C270—and one female—C002(B1)) exhibit the co-occurrence of cranial and post-cranial injuries (Tables S2 and S4). In five of these cases, there is consistency in the presence or absence of signs of healing between the cranial and postcranial skeleton. There are two exceptions: one individual with one unhealed and one healed cranial and two healed postcranial injuries (C221), and another with two unhealed cranial and one unhealed and one healed postcranial injuries (C225). Moreover, it is relevant to note that all but one (C270) of these seven individuals show arrowhead injuries in combination with other types of injuries. Another individual, while not showing the co-occurrence of cranial and post-cranial injuries, exhibits an unhealed arrowhead injury in combination with a healed fracture (male C227). Apart from those 27 individuals, there are two individuals that show a single case of unhealed cranial or postcranial injury and close association with isolated arrowheads that show use-wear signs of impact (males C230 and C234, respectively) (Table S1).

## Discussion

### The extent of violence

Trauma has proven to be elusive in skeletal remains^38^. Violent attacks often lead to soft tissue injuries and only a percentage leave traces on bone^39^. In a European Neolithic context, this percentage is estimated to be ca. 30% for arrowhead injuries and 50% for overall bodily trauma^3^. Even in cases of small- or medium-scale prehistoric massacres where skeletal preservation is good, where there is usually a relatively low number of individuals involved (generally < 35) and where it is reasonably clear that all were buried simultaneously, the percentage of individuals affected by unhealed trauma rarely exceeds 50%^3, 9^. This situation is worsened when facing large, multiple burials with extensive commingling, disarticulation and fragmentation like SJAPL. Here, the ratio of trauma detected in the present study is 23.1% (unhealed and healed injuries combined), i.e., 78 out of the 338 individuals documented at the site, which is high even for the “bloody” European Neolithic^29^, where estimated overall rates of trauma on the order of 7–17%^3^ suggest this may be the most perilous period of pre/history.

Unhealed traumas are present in 10.1% of the individuals at SJAPL, accounting for a total of 54 injuries. This prevalence is conservative and could be higher, as there are 11 isolated unhealed postcranial injuries that cannot be attributed to specific individuals. In any case, the rate is clearly elevated compared to overall crude prevalence rates of individuals with unhealed trauma estimated for the European Neolithic, which are on the order of 2–5%^3^.

Apart from the 65 unhealed traumas identified, there is evidence of striations and fractures consistent with impacts on most of the isolated arrowheads studied^20, 22^, which suggests a much larger number of victims^22^. As only about one in three arrows shot into the body are expected to strike bone and/or leave recognizable traces [e.g.,^40^] and several observations of close association between skeletal elements and projectiles were recorded in the field^19^, it does not seem unreasonable to suggest that most, if not all, of the 52 isolated arrowheads recovered at the site had impacted bodies and entered the rockshelter inside them. If, for the sake of the argument, it is assumed that each of these arrowheads inflicted or, at least, accompanied fatal injuries and that both arrowheads and unhealed postcranial injuries whose association to isolated skeletons is unknown affected individuals currently unsuspected of having suffered from trauma, this would result in 90 individuals out of 338 (26.6%) having died violently at SJAPL. The number could have been even higher taking into account that other people buried in the site may have not necessarily died in the same way, even if the burial were the result of a single violent event [cf.^16^].

In this scenario, the relatively high number of healed injuries detected (59 cranial and 30 postcranial cases affecting at least 49 individuals) and their typology, generally consistent with aggression (e.g., blunt- or sharp-force cranial trauma above the HBL, arrowhead injuries, parry fractures), suggest previous violent interactions that were not fatal, within a context of recurring conflict, over a period of at least months or more probably years. The Rioja Alavesa region of north-central Iberia, where SJAPL is located, is the area of Europe with the highest absolute number of Neolithic skeletons affected by arrowhead injuries^14, 41^, and those which have been dated are concentrated around 3380–3000 cal. BC [e.g.,^22, 42^], i.e., the same chronology attributed to SJAPL. Despite the aforementioned limitations due to the fact that the radiocarbon dates fall within the late fourth millennium cal. BC plateau in the calibration curve, this appears to confirm the likelihood of widespread conflict affecting the region at this time.

### The role of men

Males show statistically significant higher rates of trauma compared to females at SJAPL (44.9 vs. 23.9%), especially with regard to unhealed injuries (20.6 vs. 2.2%). This evidence, together with the fact that males comprise 97.6% of unhealed injuries and 81.7% of healed injuries on individuals whose sex could be estimated, suggests sex-related differences in exposure to violence. These differences, which are not mirrored in Central European Neolithic mass-fatality sites (Table 3), may indicate that a number of males acted as combatants in organised encounters, since warriorhood could have been mainly restricted to this demographic, as it is in most societies^43^. The clear predominance of adolescent and adult males in the assemblage (70%, i.e., 107 of the 153 individuals whose sex could be estimated) may also point in the same direction, as the demographic profiles of prehistoric battles^44^, contrary to those of massacres [i.e.,^10^], tend not to resemble a natural population.

**Table 3:** Demographic profile and prevalence of trauma, with detail to distribution by sex, in SJAPL and in LBK sites of Central Europe interpreted as mass-fatality events.

Site (country)	MNI	Age (%)				Sex (%)		Sex ratios	Prevalence of trauma at the site (%)			Prevalence of unhealed trauma among males documented (%)	Prevalence of unhealed trauma among females documented (%)	Unhealed injuries affecting males among sexed individuals (%)	Interpretation provided by authors	Refs.
		0–6	7–11	12–19	≥ 20	M	F		Unhealed	Healed	Unhealed and healed combined					
SJAPL (Spain)	338	28.1	10.7	17.5	40.5	31.7	13.6	2.3	10.1	14.5	23.1	20.6	2.2	97.6	Battle/s plus attritional mortality	This study
Asparn/Schletz (Austria)	67	18.7	12.7	8.2	59.0	38.8	20.1	1.9	49.3	3.0	49.3	NQ (but reported to be similar between sexes)		NQ	Massacre	^45^
Talheim (Germany)	34	20.6	17.6	9.4	50.0	29.4	20.6	1.4	61.8	14.7	64.7	80.0	57.1	60.0	Massacre	^11^
Schöneck-Kilianstädten (Germany)	26	38.5	7.7	3.8	42.3	34.6	7.7	4.5	NQ (but reported to be high)	NQ	NQ (but reported to be high)	NQ (but reported to be similar between sexes)		NQ	Massacre	^16^
Halberstad (Germany)	9	0.0	0.0	11.1	88.9	88.9	11.1	8.0	77.7	11.1	77.7	87.5	0.0	100.0	Execution	^9^

*NQ* not quantified.

The fact that 11 of 13 cases of arrowhead injuries are linked to males and that most skeletons showing association with isolated arrowheads with signs of impact are male (Table S1), suggests that men were preferentially exposed to violence at a distance. However, injuries to the head also support their participation in close-contact encounters. In SJAPL, cranial trauma is documented on all sides of the head, but the fact that injuries mainly affected lateral (n = 36) and, especially, anterior (n = 28) aspects suggests that many of those were likely inflicted from the front. However, injuries to the posterior aspect (n = 15) and parity between left and right aspects (n = 17 vs. 19), which is contrary to the expectation of a predominance of injuries to the left side resulting from between right-handed assailants, also indicate blows struck from the rear. These could have been inflicted on those fleeing or on already fallen individuals, which is also in line with the trajectories of most arrowhead injuries. The higher frequencies of both injuries per individual and injury recidivism among males also suggest that they were more likely to be exposed to re-occurring violence than other age-sex groups.

The unusually high number of adolescent males exhibiting skeletal violence at SJAPL compared to other Neolithic and Chalcolithic mass graves [e.g.,^10, 46, 47^] or to the Bronze Age battle of Tollense^44^ might suggest that they were pushed to assume a combatant role due to the scarcity of male adults resulting from a longstanding period of regional conflict and instability. The fact that this demographic shows the highest frequency of unhealed injuries per individual (2.2) could reflect either their temerity and/or their more limited training and ability in combat. Potentially, the loss of young adult males in battles and raids would have been seen by opponents as an indicator of the increased vulnerability of the surviving community to aggression [cf.^22^].

### The role of women, children and infants

However, the existence among adolescent and adult females and children and infants of instances of both unhealed and healed trauma indicates that not only adolescent and adult males were exposed to violence at SJAPL. The involvement of females and children also occurs in other European Neolithic contexts [e.g.,^3, 48^].

The temptation is to assume that those adolescent and adult females, children and infants were killed similarly to males or, alternatively, abducted. While it is not impossible that some women and older children participated in violent confrontations, in SJAPL much lower frequencies than males have been identified for these population segments, particularly regarding unhealed trauma (2.2% in females and 5.3% in children and infants), than in Neolithic massacres (Table 3). Moreover, available skeletal evidence for violence in these groups mostly focuses on healed trauma, which may relate to both intra- and inter-group violence^36, 49^. Regarding the former, the incidence of conflict has been associated with an intra-group preference for rearing male children, due to their higher value in warlike societies, along with female infanticide or neglect, and harsh treatment and abuse of women^43, 50^. Simultaneously and/or alternatively, boys are usually reared to instil aggressive personality traits among future warriors, which may include severe corporal punishments^51^. Regarding the latter, women and children are easy and frequent targets in inter-group conflict. They may be slaughtered in violent attacks. Women and girls are also especially vulnerable to sexual assault during large-scale conflicts^52^. Defensive injuries among victims from these assaults may be occasionally found mainly in bones of hands, forearm and even upper arm, and eventually legs and feet^53^. However, only about a dozen, mostly healed fractures observed in SJAPL to hands and forearms are compatible with defence wounds, and they are not restricted to this demographic. Women and children can also be enslaved. Indeed, kidnapping has been proposed as a reason behind the scarcity of young females and, occasionally, infants in a number of conflict-related Neolithic mass graves [e.g.,^10^], being a useful mechanism for simultaneously decreasing the size and the renewal capacity of enemy groups and increasing that of one’s own. However, in SJAPL adolescent and adult females are similarly represented in all ages categories and no demographic bias against young females has been detected. Thus, the most likely explanation is not that adolescent and young females were underrepresented here, but rather that adolescent and adult males are overrepresented.

### The nature of the assemblage

In the light of the new skeletal findings and the aforementioned evidence, it is not inconceivable that SJAPL includes a large number of casualties of violence, potentially in the form of one or more “war layers” resulting from battles and/or raids where the involvement of males was dominant. Previous demographic modelling concluded that the assemblage was the result of violence-related events (massacres excluded) in combination with attritional mortality^22^. Unfortunately, only a limited number of demographic scenarios have been tested so far using the Population & Cemetery Simulation software^54^, and the weight of violent events and of attritional mortality in the formation of the assemblage remains unknown. However, demographic evidence of overrepresentation in age categories less likely to die naturally (namely older children and adolescents) in SJAPL may support an important role of mass mortality events, since those age categories do not usually show a surplus in populations affected by re-occurring small-scale violent events [e.g.,^55^]. Also, the relatively low prevalence of injury recidivism (i.e., the co-occurrence of unhealed and healed trauma) at SJAPL may point to this direction^55^.

**Figure 6:**
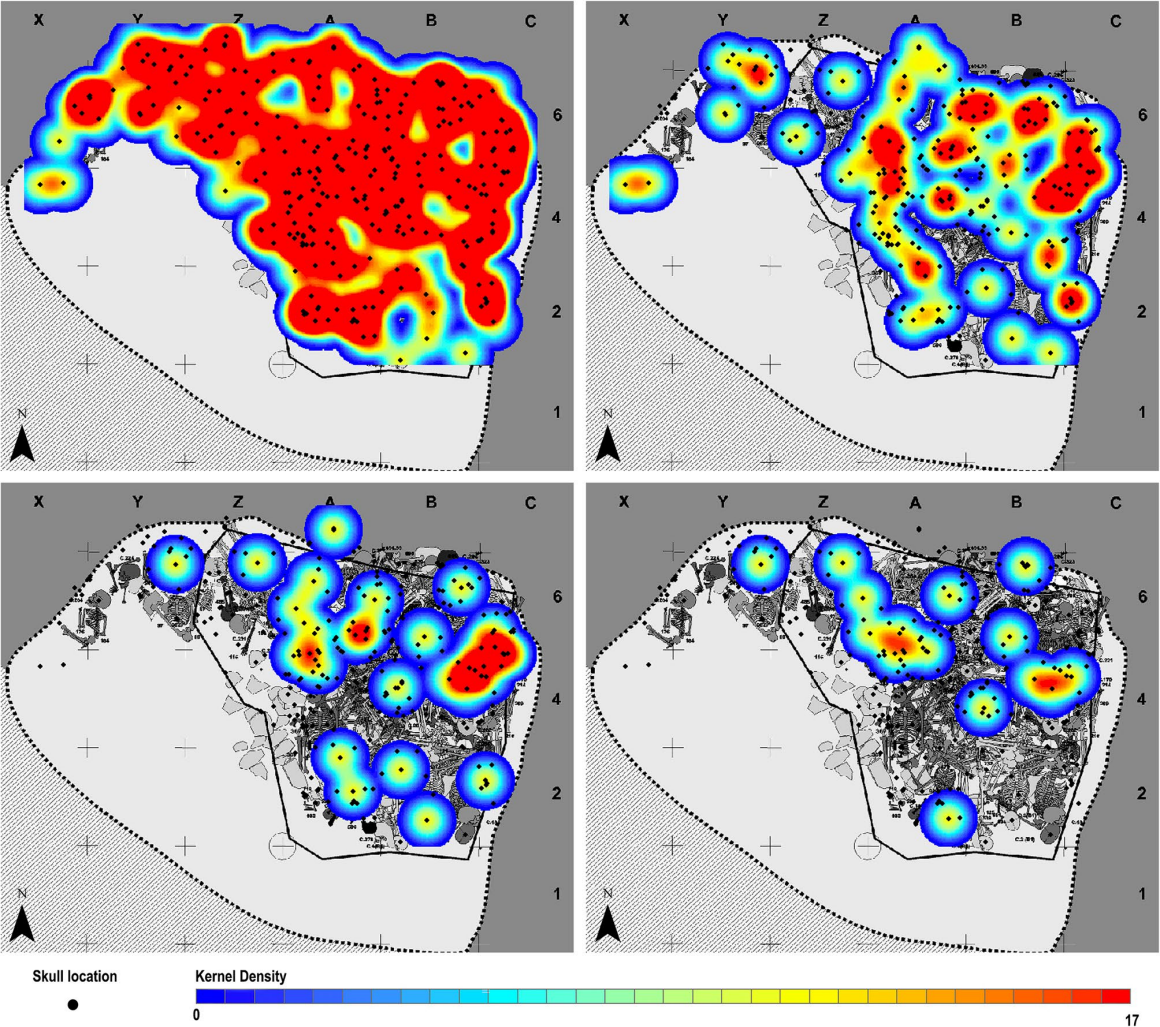
Kernel quadratic density analysis (ArcGIS 10.2, based on Silverman’s equation 4.5^56^) of the location of the crania with recorded x/y coordinates from all the individuals (top left), from individuals with both unhealed and healed trauma (top right), from individuals with unhealed trauma (bottom left), and from individuals with unhealed arrowhead injuries and/or in close association with isolated arrowheads with signs of having impacted bodies (bottom right). The exact location of all the crania, corresponding to the central point of their vaults and here represented with black dots, is shown in every panel for comparison.

Spatial analysis of the crania of those individuals affected by trauma at SJAPL does not provide any recognisable patterning suggesting the potential location of specific “war layers”, whether or not individuals with healed injuries are included (Fig. 6). Nor do field data regarding the superimposition of connected skeletons (in the absence of depth records) support that individuals affected by unhealed trauma, potentially a fourth of the deposit, were buried in different stages of the site’s mortuary use^22^.

The remains of those who die in violent encounters tend to be either left unburied or be buried very close to where they fall or where they later succumb to their injuries. Individuals killed and buried at SJAPL are likely to have been mainly defenders in a scenario wherein their settlement was attacked or their territory or resources raided. Recovering members of a raiding party or larger force, killed at a location away from their home territory and bringing them back for burial would be challenging. In any case, the evidence that many individuals killed were buried at SJAPL indicates, first, that the location of encounter/s was not far away from the site and, second, that such defense was essentially successful or, at least, that there were sufficient members of the community surviving to bury them.

### Wider impacts

In war, conflict-related casualties are not always a result of physical violence. Settlements can be raided, food stores plundered, fields burned, livestock stolen and daily labour activities and trade disrupted^57, 58^. As a result, some individuals buried at SJAPL could have been affected by deprivation such that they succumbed to illness and starvation, particularly infants, elderly and those health-compromised. Evidence for non-specific stress indicators and nutritional deficiencies recorded in SJAPL during the osteological re-examination may be interpreted as a glimpse of the health and environmental costs of warfare, particularly of food scarcity and malnourishment. The rates of *cribra orbitalia* detected here (36.7%) (Table 4) are unusually high compared to those generally estimated for Iberian Late Neolithic and Chalcolithic sites (generally 2–3%)^59^. And they largely double those estimated for the end of the Central European LBK (15.4%)^60^, where a degree of resource scarcity within a context of endemic violence seems to have occurred, although higher rates have been reported for some violence-related sites of the period like Asparn/Schletz in present-day Austria (54.0%)^45, 60^. The fact that rates in SJAPL seem to decrease while age increases (from 88.5% in infants to 17.9% in adults), suggests a potential decrease in life quality within a short time span. Conversely, in Asparn/Schletz, the more elevated prevalence in adults (67%) than in non-adults (42%)^60^ seems more difficult to conciliate with a sudden and generalized worsening of life conditions, where infants –due to faster bone turnover rates– should theoretically show a higher prevalence. Linear enamel hypoplasia rates are also high in SJAPL (53.1%) (Table 4), especially compared to those reported for other broadly coeval Iberian sites (generally < 10%)^59, 61^, while similar to those estimated for the end of the LBK (54.2%)^60^. Prevalence seems to be higher among men (63.3%), and in this case is more consistent throughout age classes (Table 4). Unfortunately, the variable preservation of many skulls precluded systematically recording *cribra cranii*. Moreover, skeletal changes attributable to scurvy (a disease associated to prolonged vitamin C deficiency) have been identified on six infants, which suggests poor micronutrient intake potentially linked to a degree of food scarcity. In this regard, the finding of three and 13 long bone deformities attributable to rickets (a disease associated to vitamin D, calcium and/or phosphorus deficiency) in individuals interred in the nearby and broadly coeval megalithic graves of Alto de la Huesera and Longar, respectively, and of Harris lines in two out of five tibiae x-rayed in the latter, may also be interpreted as a possible result of problems with food availability and supply in the region during the Late Neolithic^62, 63^.

**Table 4:** Frequencies of *cribra orbitalia* and linear enamel hypoplasia recorded in SJAPL (this study).

Indicator	Frequency	Frequencies by age				Frequencies by sex	
		0–6	7–11	12–19	≥ 20	M	F
*Cribra orbitalia*^a^	81/221	23/26	16/38	23/44	17/95	23/85	7/34
Linear enamel hypoplasia^b^	43/81	5/10	1/5	12/22	25/44	28/44	5/14

^a^Frequencies are reported based on the presence of indicators against the number of skulls/individuals preserving at least one orbit.

^b^Frequencies are reported based on the presence of indicators against the number of skulls/individuals preserving anterior teeth from either maxillae or mandibles.

Isotopic evidence for restricted landscape use and territoriality in the region has been suggested to reflect both a preoccupation with protecting economic resources in a scenario of increasingly overlapping catchment areas due to population increase, and reduced intergroup interaction due to perceived danger and insecurity^64^. In this regard, the existence in the region of large communities, potentially including that using SJAPL for burial, can be satisfactorily explained in purely economic terms (e.g., fertile lands, hypersaline lakes potentially providing salt, strategic geographic position), but an additional driver may be the threat of warfare, as smaller groups would be at higher risk of annihilation^65^.

Neolithic warfare has traditionally been assumed to be curtailed by the supposedly limited economic and logistic capabilities of early agrarian societies, since a large surplus would have been necessary to support sustained large-scale conflict. Therefore, it has been generally characterized as a mixture of rapid assaults or short raids, generally lasting no more than a few days and affecting no more than 20 or 30 individuals. The exception might be some causewayed enclosures in southern Britain, such as Crickley Hill and Hambledon Hill, which—despite scarce evidence of skeletal trauma—show signs of co-ordinated assaults by apparently large groups^66^; and, although more debatable, the aforementioned Austrian site of Asparn/Schletz^15, 45^. There, a minimum of 67 incomplete individuals were found scattered in the base of a ditch. Skulls (n = 33), representing all ages and both sexes, provided evidence of unhealed trauma. As twelve of these individuals were dated towards the collapse of the settlement, ca. 5210–4950 cal. BC, the human remains were attributed to a single massacre responsible for the final demise of village^45^ and which may have tentatively involved up to 200 individuals considering that ca. 80% of the ditch remains unexcavated. However, skeletal evidence recovered from other coeval Central European LBK ditches (e.g., Vráble, Herxeim) may point to complex ritualized cumulative practices at the end of the period, where violence may or may not be implicated^67^. This is definitely not the case at SJAPL, where the potential duration (at least months if not years) of conflict, the number of direct casualties, the male-biased demographic, and the possible social and economic outcomes (i.e., population sizes involved, health costs, food scarcity, restricted mobility) identified in Late Neolithic Rioja Alavesa region, suggest significant wider impacts than previously appreciated in the European record until over a millennium later [e.g.,^44^].

### Potential causes and actors

The reasons behind such a conflict are so far unclear. Variability in mortuary practices and/or burial sites (i.e., caves and rockshelters *vs.* monuments) present in the region during the Late Neolithic has been suggested to reflect cultural alterity, which would explain differences detected in subsistence strategies and landscape use between both groups^64^. It is possible that divergent beliefs and lifestyles could have been a source of tension and competition, escalating into lethal violence. However, diversification in funerary treatment may also be seen as the culmination of internal unrest as a means to negotiate social conflict and community boundaries [e.g.,^68^]. Unfortunately, settlements and associated material culture are virtually unknown in the Rioja Alavesa region. Grave goods are also scarce and undiagnostic. Formal weapons are absent and arrowheads found in SJAPL and in nearby sites with skeletal signs of violence including arrowhead injuries such as Longar monument, Las Yurdinas II swallet and La Peña de Marañón rockshelter, have shown notable intra-site variability in both shape (mainly foliated and rhomboidal styles) and size^14, 20, 41, 42^ with no diachronic patterning, which has also been described in many coeval violence-related contexts across Iberia^69^. These features limit finer-scale characterization of the conflict and its temporality, main causes and actors. The possible arrival of people to the region during this period, suggested by some authors based on the occupation of foothill areas, the emergence of burial in caves and rockshelters, and the appearance of new types of megalithic graves and ritual changes^70^, may have provided a possible trigger. However, available isotopic analyses of Late Neolithic human individuals from the region suggest that most were local^64^, and aDNA studies, despite their limits, do not support significant inputs from genetically distant populations^71^. This may tentatively suggest endogenous growth and other internal factors as the most plausible causes behind those novelties and, potentially, the roots of conflict.

## Conclusions

The distinction between unhealed and healed occurrences is of the highest importance when interpreting trauma in the skeletal record of mortuary sites^72^. This is particularly relevant in cases where a large number of individuals are involved and the precision of absolute dating methods is limited, as in the Late Neolithic rockshelter of SJAPL (north-central Iberia) studied here.

A re-examination of the skeletal assemblage comprising a minimum of 338 individuals has provided evidence of 65 unhealed and 89 healed injuries, which combined affect ca. 45% of adolescent and adult males recorded at the site. The fact that most unhealed injuries recorded in sexed individuals affect men suggests that many males acted as combatants and eventually died in battle and raids. This, together with the identification of at least 41 isolated arrowheads likely having impacted into tissues and the haphazard and interwoven superimposition of many bodies, may indicate that a large number of the individuals deposited in SJAPL—including mainly men, but also women and children– were involved in conflict.

It is possible that the existence, in a context of high population pressure, of different cultural groups with distinct lifeways and funerary practices was a source of tension and competition and, as a result, a major catalyst of lethal violence. Evidence of arrowhead injuries and other skeletal signs of violence (e.g., cranial trauma, parry fractures) in other coeval sites of the region, supports an image of standing and organized violence between rival communities. This, together with skeletal and isotopic evidence compatible with biological stress and malnourishment and with fixed mobility in the region, suggests wider social impacts to an extent that has not previously been seen for the European Neolithic record.

## Materials and methods

### Materials

All human skeletal remains from SJAPL have been re-examined for this study. The collection is held in the Bibat Museum of Álava (Basque Country, Spain) and stored under the ‘SJAPL’ general inventory number in boxes numbered RRCC.02211-03312.1 to 145, except for skulls C15(B1) and C123, which are included in the museum’s permanent exhibition.

Preservation is generally good, although elements show high fragmentation, particularly crania. Despite many crania having been carefully reassembled where possible to facilitate previous osteological analyses^19^, a substantial number are only partially preserved (10.1% with 100–75% of cranium preserved, 19.8% with 75–50%, 32.5% with 50–25%; 37.6% with < 25%). Facial and basilar regions are often missing as a result of their intrinsic fragility. Instances of postmortem and recent fractures are also frequently seen in long bones, pelvic bones, scapulae, ribs and vertebral arches, particularly in *loci minoris resistentiae*. Severe sediment compression due to the fallen roof block and, secondarily, recent manipulation (controlled explosion of the collapsed roof, excavation, laboratory analysis), are the most important taphonomic agents. Fragmentation, disarticulation and dispersion have been described as greater in the uppermost layer^23^. Long bone metaphyses and epiphyses, scapular bodies, ribs shafts, vertebral arches and small bones of the hands and feet are generally poorly represented or compromised by postmortem and recent fractures. Micro-striations as a result of internal displacement and rodent tooth marks are occasionally observable on bone.

The large number of individuals deposited in SJAPL, together with the high compression, fragmentation and superimposition that the human remains showed during fieldwork and the lack of precedents with contrasted methodological protocols in recording similar assemblages, made the in situ documentation of skeletal connections and provenience a very complex task. Thus, only 278 crania and 90 skeletons were given horizontal coordinates and/or recorded in drawings and so can be virtually relocated. However, depths are unknown in all cases and only superimposition relationships among complete skeletons can be established with the available spatial data. Moreover, the fact that articulated skeletons or skeletal assemblages have not been kept within the same boxes, that cranial and postcranial elements follow different numeration series (even when attributed to the same individual), and that many postcranial bones have lost their inventory numbers over time, have worsened the situation such that the recovery of skeletal connections for individual trauma recording is not possible in many cases. As a consequence, a large part of the collection has been treated as a skeletal assemblage mainly composed of isolated bones.

### Age and sex estimation

Morphognostic methods were used in age and sex estimation. Age estimation was based on skeletal and dental development, maturation, wear and degeneration of the available skeletal elements^73^. Adolescent and adult sex estimation primarily made use of cranio-mandibular indicators^74, 75^ and, where possible in connected individuals, of dimorphic pelvic features^73^.

### Analysis of trauma

Trauma was assessed with reference to the standards of the field^38,76–78^. Macroscopic examination of bone surfaces, aided by the use of low-magnification lenses, was used to closely examine fracture margins^79^. Morphological and metric data were recorded. Healed injuries (i.e., antemortem, those inflicted some time before the death) were identified upon detection of fractures showing signs of bone remodeling. Unhealed injuries (i.e., perimortem, those that occurred around the time of death and, therefore, do not show bone remodeling) were differentiated from postmortem fractures (i.e., those occurred after the deposition of the skeleton) through analysis of the following features: (1) outline: linear, depressed or stellate (for crania) and longitudinal, transverse, curved or V-shaped (for the postcranium); (2) angle: right, oblique or mixed; (3) edge: smooth or jagged; and (4) presence or absence of some observations, including: beveling, comminuting, conchoidal scars, adhering flakes, peeling and patination^79^. Location of injuries was recorded using Hussain et al.’s zonation method^32^. Types of injuries were classified in blunt-force, sharp-force and penetrative traumas following Smith’s definitions^29^, and in a “other” category when they did not match those definitions (e.g., bone dislocations). Determinations of the temporal sequence of blunt-force traumas to the skull were conducted following Puppe’s rule^33^.

### Analysis of non-specific indicators of stress and nutritional deficiencies

Non-specific skeletal stress indicators (particularly, *cribra orbitalia* and linear enamel hypoplasia) and pathological features compatible with nutritional deficiencies (e.g., scurvy, rickets) were recorded based on standard criteria for the identification of such lesions^77^. Frequencies are reported based on the presence of indicators against skulls that preserved areas where these are generally recorded, i.e., orbits in the case of *cribra orbitalia* and anterior teeth of either maxillae or mandibles in that of linear enamel hypoplasia (Table 4). For nutritional deficiencies, differential diagnosis relies on bony lesions recorded on the cranium and, when associated, the postcranium.

### Statistical tests

2 × 2 contingency *χ*^2^ tests^80^ were used to assess differences in prevalence of trauma (e.g., between aspects of crania affected by trauma or between males and females affected by trauma). Effect size was assessed using Cramér’s *V*^81^.

### Supplementary Information

Supplementary Information.

## Data Availability

All data generated or analysed during this study are included in this published article [and its supplementary information files].
