# Supplementary material for: Large-scale violence in Late Neolithic Western Europe based on expanded skeletal evidence from San Juan ante Portam Latinam
Source: Sci Rep. 2023 Nov 2;13:17103. doi: 10.1038/s41598-023-43026-9 (PMC10622514; doi:10.1038/s41598-023-43026-9)
Supplement: Supplementary file 1 — Supplementary Information. [file 41598_2023_43026_MOESM1_ESM.pdf]

Large-scale violence in Late Neolithic Western Europe based on expanded skeletal evidence from San Juan ante Portam Latinam

Teresa Fernández-Crespo, Javier Ordoño, Francisco Etxeberria, Lourdes Herrasti, Ángel Armendariz, José I. Vegas & Rick J. Schulting

SUPPLEMENTARY INFORMATION

Table S1. List of arrowheads documented at SJAPL, including those found isolated and those embedded in bones (i.e., arrowhead injuries[1]), with reference to use-wear signs of impact and, when available, their association to bones/skeletons, impact area, signs of healing and age and sex of the individuals [after 19,20,21].

Arrowhead no.	Impact signs	Type of impact sign	Association	Associated skeleton/assemblage	Impact area	Healing	Age [6]	Sex [7]
8	YES	Impact striation	YES	Unnumbered	Upper limb		?	?
13	? [2]		NO					
15	? [2]		NO					
17	YES	Impact striation & flute-like fracture	NO					
19	YES	Flute-like fracture	YES	Unnumbered	Unrecorded		?	?
34	YES	Flute- and burin-like fracture	NO					
36	YES	Embedded in bone	YES	36B	Right coxal bone	Yes	IA	M
38A	? [3]		NO					
41	YES	Flute-like fracture	NO					
46	YES	Flute-like fracture	NO					
52	YES	Flute- and burin-like fracture	NO					
53	YES	Impact striation & flute-like fracture	NO					
55	NO		NO					
56	NO		NO					
63	? [3]		NO					

64	? [3]		NO					
65	YES	Flute-like fracture	NO					
504	YES	Impact striation	NO					
511	YES	Embedded in bone	YES	C.161	1st lumbar vertebra	No	YA	M
513	YES	Burin-like fracture	YES	C.177	Thorax		ADOL	?
515	YES? [2,4]	Distal end broken	NO					
516	YES	Embedded in bone	YES	C.225	Left coxal bone	No	MA	M
523	YES	Embedded in bone	YES	C.198	Thorax	No	ADOL	M
526	? [2]		NO					
527	YES	Flute-like fracture	NO					
528	YES	Embedded in bone	YES	C.218	Left scapula	No	ADOL	M
529	YES	Flute-like fracture	YES	C.218	Unrecorded		ADOL	M
532	YES	Impact striation	NO					
535	YES	Embedded in bone	YES	C.123	Neck	Yes	YA	M
607	YES? [2,4]	Distal end broken	YES	C.225	Neck		MA	M
612	YES	Burin-like fracture	YES	C.230	Abdomen (right coxal bone)		YA	M
613	YES	Embedded in bone	YES	C.227	8th dorsal vertebra	No	YA	M
618	YES	Flute-like fracture	NO					
619	YES? [4]	Distal end broken	YES	C.234	Thorax/abdomen		YA	M
620	YES	Burin-like fracture	YES	C.227	Forearm		YA	M
621	YES	Flute-like fracture	YES	Unnumbered	Thorax		?	?
622	YES	Flute-like fracture	NO					
629	YES	Impact striation & flute-like fracture	YES	Unnumbered	Thorax (vertebrae)		?	?
632	? [3]		YES	Unnumbered	Left thigh		IA	M

642	NO		YES	C.260	Right elbow		MA	M
643	YES? [2,4]	Distal end broken	YES	C.260	Left hemithorax		MA	M
644	YES	Flute-like fracture	YES	Unnumbered	Knee		IA	M
645	NO		NO					
651	NO		YES	C.295	Head		C	M?
654	YES	Impact striation & flute-like fracture	NO					
655	? [3]		NO					
658	YES	Embedded in bone	YES	C.221	Left coxal bone	Yes	ADOL	M
663	YES? [2,4]	Distal end broken	YES	C.299	Head		C	?
665	YES? [2,4]	Distal end broken	NO					
668	YES? [2,4]	Distal end broken	YES	C.128	Abdomen (right coxal bone)		MA	M
671	YES	Flute-like fracture	NO					
672	YES	Embedded in bone	YES	C.212	Radius and ulna	Yes	YA	M
673	YES? [2,4]	Distal end broken	YES	Unnumbered	Right leg		IA	M
674	YES	Impact striation	YES	SJAPL 503-504	Left hemithorax		IA	?
675	YES? [2,4]	Distal end broken	YES	C.212	Thorax (lumbar vertebrae)		YA	M
676	NO		YES	C.298	Head		I	?
678	YES	Impact striation & flute-like fracture	YES	C.331	Abdomen		MA	M
695	YES	Embedded in bone	YES	C.2(B1)	Dorsal vertebra	Yes	ADOL	F?
696	YES? [2,4]	Distal end broken	NO					
697	YES? [4]	Distal end broken	NO	C.331	Unrecorded		MA	M
706	? [3]		NO					
893	? [2]		YES	C.318	Abdomen (left coxal bone)		MA	M

Unknown [1]	YES	Injury preserved, no arrowhead found	YES	C.122B	Frontal bone	Yes	ADOL	M
Unknown [1]	YES	Injury preserved, no arrowhead found	YES	C.225	Left rib	Yes	MA	M
Unknown [1]	YES	Injury preserved, no arrowhead found	YES	SJAPL 871	Right tibia	Yes	ADOL	M
Unknown [1]	YES? [5]	Injury preserved, no arrowhead found	YES	SJAPL Z	Rib	Yes	?	?

[1] Cases of arrowhead injury whose arrowheads could not be found/identified during the excavation are also listed in the table to include all the archaeological evidence potentially related with arrowhead-based violence.
[2] No available use-wear analysis.
[3] Bad preservation did not allow use-wear diagnosis.
[4] Distal end/ tip breakage is interpreted as sign of potential impact.
[5] Doubtful case of arrowhead injury. May be compatible with another skeletal lesion. Another case that was considered in previous works as a doubtful arrowhead injury in a skull (C.184) [after 19], has been dismissed as a traumatic lesion during the course of the re-examination and, therefore, is not included in the table.
[6] Where: *I* = infant (0–6 years of age); *C* = child (7–12); *ADOL* = adolescent (12–19); *YA* = young adult (20–39); *MA* = middle adult (40–59); *OA* = old adult (>60); *IA* = indeterminate adult (>20).
[7] Where: *M* = male; *M?* = probable male; *F* = female; *F?* = probable female; *?* = ambiguous/indeterminate.

Table S2. Cranial injuries identified at SJAP in this study, by individual.

Cranium	Age[1]	Sex[2]	No. injuries	Injury ID	Location[3]	Probability	Fracture description	Max. diameter (cm)	Injury type[4]	Healing	TSB[5]	Figure	Source
C001(Z4)	MA	M	2	C001(Z4)-1	16	Clear	Oval, depressed, internal bevel	> 2	BFT	No	1	4a, 4b	This study
				C001(Z4)-2	16	Clear	Oval, depressed, internal bevel	> 1.5	BFT	No	2	4a, 4b	This study
C002	YA	M?	1	C002-1	**39**, 17, 18, 32, 33, 35, 37	Clear	Irregular, depressed, internal bevel, radiating secondary and tertiary fracture lines	4.0	BFT	No		4c	This study
C002(B1)	ADOL	F?	1	C002(B1)-1	17	Clear	Oval, depressed, only external table affected	1.3	BFT	Yes			[23:appendix], This study
C002(Y4)	ADOL	F	1	C002(Y4)-1	35	Clear	Oval, depressed, only external table affected	4.5	BFT	Yes			[19], This study

C003(B1)	YA	M	2	C003(B1)-1	12	Clear	Irregular, depressed, radiating secondary fracture lines	NA	BFT	No	1?		This study
				C003(B1)-2	15	Clear	Linear fracture, radiating secondary fracture lines	NA	BFT	No	2?	4d	This study
C009(B1)	I	?	1	C009(B1)-1	18	Clear	Circular, depressed, only external table affected	0.5	BFT	Yes			[23:appendix], This study
C011(Y4)	C	?	1	C011(Y4)-1	38	Probable	Oval, depressed, internal bevel	> 2.5	BFT	No			This study
C012-014(A1)	OA	F	1	C012-014(A1)-1	18	Clear	Circular, depressed, only external table affected	1.9	BFT	Yes			[19], This study
C015(B1)	MA	M	5	C015(B1)-1	17	Clear	Circular, depressed, only external table affected	0.4	BFT	Yes			[19], This study
				C015(B1)-2	36	Clear	Circular, depressed, only external table affected	0.4	BFT	Yes			[19], This study
				C015(B1)-3	28	Clear	Circular, depressed, only external table affected	0.4	BFT	Yes			[19], This study
				C015(B1)-4	35	Clear	Circular, depressed, only external table affected	0.4	BFT	Yes			[19], This study
				C015(B1)-5	39	Clear	Circular, depressed, only external table affected	0.4	BFT	Yes			[19], This study
				C015(B1)-6	28	Clear	Oval, depressed, only external table affected	1.0	BFT	Yes			This study
C023(Z4)	IA	?	1	C023(Z4)-1	28	Clear	Trepanation by drilling, both tables affected, partially healed	0.4	TRE	Yes			[19], This study
C107	YA	M	1	C107-1	37	Clear	Circular, depressed, only external table affected	1.3	BFT	Yes			[19], This study
C111	YA	M	1	C111-1	31	Clear	Oval, depressed, only external table affected	1.2	BFT	Yes			[19], This study
C122b	ADOL	M	1	C122b-1	14	Clear	Oval, depressed, deep, both tables affected, partially healed	0.6	PEN	Yes			[19], This study
C123	MA	M	1	C123-1	44	Clear	Arrowhead embedded, reactive bone lesion below occipital bone	NA	PEN	Yes			[19], This study
C129	C	?	2	C129-1	14	Clear	Circular, depressed, only external table affected	1.4	BFT	Yes			[19], This study
				C129-2	32	Clear	Irregular, depressed, only external table affected	1.6	BFT	Yes			This study
C130	C	?	1	C130-1	**1**, 14	Clear	Linear fracture, right, smooth	NA	BFT	No		4e	This study
C133	MA	F	1	C133-1	16	Clear	Circular, depressed, only external table affected	1.7	BFT	Yes			[23:appendix], This study

C137	YA	F	1	C137-1	**33**, 26	Probable	Oval, depressed, radiating fracture lines	> 2.5	BFT	No			This study
C141b	ADOL	M	2	C141b-1	16	Clear	Oval, depressed, only external table affected	1.3	BFT	Yes			[19], This study
				C141b-2	**17**, 18	Clear	Oval, depressed, only external table affected	1.1	BFT	Yes			This study
C148	I	?	1	C148-1	**32**, 27, 37	Clear	Oval, circling, depressed, internal bevel	12.1	BFT	No		4f	This study
C153	MA	M	1	C153-1	18	Clear	Oval, depressed, only external table affected	1.1	BFT	Yes			[19], This study
C156	I	?	1	C156-1	**34**, 16	Probable	Oval, depressed, radiating secondary fracture lines	> 3	BFT	No			This study
C166	YA	M	3	C166-1	35	Clear	Circular, depressed, only external table affected	1.1	BFT	Yes			[19], This study
				C166-2	37	Clear	Circular, depressed, only external table affected	0.9	BFT	Yes			This study
				C166-3	32	Clear	Linear, depressed, only external table affected	0.8	SFT	Yes			This study
C168	MA	M?	2	C168-1	**33**, 32	Probable	Linear, depressed, internal bevel, adhered bone flakes	> 5	SFT	No		4g, 4h	This study
				C168-2	**1**, 14	Probable	Linear fracture, right, smooth, internal bevel	NA	BFT	No			This study
C169	YA	M	1	C169-1	**36**, 37	Clear	Irregular, depressed, internal bevel, radiating secondary and tertiary fracture lines	2.8	BFT	No		4i	This study
C171	MA	M	2	C171-1	13	Clear	Circular, depressed, only external table affected	0.7	BFT	Yes			[19], This study
				C171-2	31	Clear	Circular, depressed, only external table affected	0.7	BFT	Yes			This study
C172	ADOL	M	3	C172-1	37	Clear	Irregular, depressed, internal bevel, radiating secondary fracture lines	NA	BFT	No			This study
				C172-2	**1**, 14	Clear	Linear fracture, right, jagged, internal bevel	NA	BFT	No			This study
				C172-3	**28**, 31, 32	Clear	Punctuated, radiating secondary fracture lines	NA	BFT	No		4j	This study
C178b	ADOL	M	1	C178b-1	16	Clear	Circular, depressed, only external table affected	0.6	BFT	Yes			[19], This study
C188	ADOL	M	2	C188-1	12	Clear	Oval, depressed, only external table affected	1.1	BFT	Yes			[19], This study

				C188-2	**12**, 13	Probable	Linear fracture, radiating secondary fracture lines	NA	BFT	No			This study
C189	ADOL	M	1	C189-1	26	Clear	Circular, depressed, internal bevel, radiating secondary and tertiary fracture lines, adhered bone flakes	4.5	BFT	No		4k	This study
C190	C	?	2	C190-1	17	Clear	Oval, depressed, only external table affected	1.5	BFT	No			[19], This study
				C190-2	39	Clear	Punctuated, depressed, radiating secondary fracture lines	NA	BFT	No			This study
C198	ADOL	M	3	C198-1	**35**, 33	Clear	Oval, depressed, internal bevel, radiating secondary fracture lines	> 4	BFT	No	1?	4l	This study
				C198-2	32	Clear	Punctuated, depressed, radiating secondary fracture lines	NA	BFT	No	2?		This study
				C198-3	14	Clear	Linear fracture, right, smooth	NA	BFT	No	NA		This study
C199	YA	M	3	C199-1	12	Clear	Oval, depressed, internal bevel	> 2.6	BFT	No	1	4m, 4n	This study
				C199-2	12	Clear	Oval, depressed, internal bevel, radiating secondary fracture line, adhered bone flakes	> 3.8	BFT	No	2	4m, 4n	This study
				C199-3	16	Clear	Oval, depressed, internal bevel, adhered bone flakes	> 4.1	BFT	No	NA		This study
C201	I	?	1	C201-1	18	Clear	Irregular, depressed, only external table affected	0.5	BFT	Yes			This study
C202/220b	ADOL	M	1	C202/220b-1	**16**, 17	Clear	Circular, depressed, only external table affected	2.0	BFT	Yes			This study
C207	YA	F	1	C207-1	32	Clear	Oval, depressed, only external table affected	2.0	BFT	Yes			This study
C208	MA	M	1	C208-1	37	Clear	Oval, depressed, only external table affected	3.1	BFT	Yes			This study
C212	YA	M	2	C212-1	**37**, 18	Clear	Circular, depressed, both tables affected	2.7	BFT	Yes			[19], This study
				C212-2	4	Clear	Oval, fracture callus	1.8	BFT	Yes			This study
C213	MA	M	2	C213-1	12	Clear	Circular, depressed, only external table affected	0.5	BFT	Yes			This study
				C213-2	31	Clear	Circular, depressed, only external table affected	1.5	BFT	Yes			This study
C218	ADOL	M	2	C218-1	15	Clear	Linear fracture, oblique, smooth	NA	BFT	No	1		This study

				C218-2	**13**, 6	Clear	Linear fracture, right, jagged	NA	BFT	No	2		This study
C221	ADOL	M	2	C221-1	9	Clear	Linear fracture, right, smooth	NA	BFT	No			This study
				C221-2	39	Clear	Circular, depressed, only external table affected	0.8	BFT	Yes			This study
C225	MA	M	2	C225-1	33	Clear	Oval, depressed, internal bevel, radiating secondary fracture lines	> 4	BFT	No	1	4o, 4p	This study
				C225-2	32	Clear	Oval, depressed, internal bevel, radiating secondary fracture lines	> 3.8	BFT	No	2	4o, 4p	This study
C229	ADOL	M	2	C229-1	30	Clear	Oval, depressed, internal bevel	> 4.1	BFT	No	1		This study
				C229-2	**31**, 28	Clear	Oval, depressed, internal bevel	> 6.5	BFT	No	2	4q	This study
C230	YA	M	1	C230-1	38	Clear	Oval, depressed, internal bevel, radiating secondary fracture lines	4.6	BFT	No			This study
C232	MA	M	1	C232-1	33	Clear	Oval, depressed, only external table affected	1.4	BFT	Yes			[19], This study
C233	YA	M	1	C233-1	16	Clear	Healed (?) scalp injury, only external table affected	1.5	SFT	Yes			[19], This study
C236	MA	F	1	C236-1	39	Clear	Circular, depressed, only external table affected	2.0	BFT	Yes			[19], This study
C240	YA	F	1	C240-1	35	Clear	Circular, depressed, only external table affected	0.5	BFT	Yes			[19], This study
C242	MA	M	2	C242-1	35	Probable	Drop-like, depressed, internal bevel, radiating secondary fracture lines, adhered bone flakes	3.8	BFT	No			This study
				C242-2	37	Clear	Oval, depressed, only external table affected	0.7	BFT	Yes			This study
C244	I	?	1	C244-1	36	Probable	Oval, depressed, internal bevel, radiating secondary fracture lines, adhered bone flakes	3.9	BFT	No			This study
C259	YA	M	1	C259-1	30	Clear	Bump, oval	1.2	BFT	Yes			This study
C261b	YA	M	4	C261b-1	32	Clear	Circular, depressed, only external table affected	2.7	BFT	Yes			[19], This study
				C261b-2	37	Clear	Circular, depressed, only external table affected	2.5	BFT	Yes			[19], This study
				C261b-3	35	Clear	Circular, depressed, only external table affected	2.6	BFT	Yes			[19], This study

				C261b-4	13	Clear	Circular, depressed, only external table affected	1.2	BFT	Yes			[19], This study
C270	MA	M	1	C270-1	17	Clear	Circular, punctuated, depressed, only external table affected	1.3	BFT	Yes			[19], This study
C271	MA	?	2	C271-1	37	Probable	Oval, depressed, internal bevel	> 3.5	BFT	No			This study
				C271-2	36?	Probable	Oval, depressed, internal bevel	> 2.9	BFT	No			This study
C272/273	YA	M	2	C272/273-1	15	Clear	Linear fracture, oblique, jagged	NA	BFT	No			This study
				C272/273-2	36	Clear	Oval, depressed, internal bevel, radiating fracture line	> 1.3	BFT	No			This study
C281	ADOL	?	1	C281-1	**13**, 14	Clear	Linear, depressed, only external table affected	2.0	SFT	Yes			[19], This study
C297	YA	M	1	C297-1	**32**, 33	Clear	Trepanation by drilling, only external table affected	1.0	TRE	Yes			[19], This study
C304	MA	M	1	C304-1	15	Clear	Circular, depressed, only external table affected	1.5	BFT	Yes			[19], This study
C306b	I	?	1	C306b-1	18	Clear	Oval, depressed, internal bevel	> 2.5	BFT	No			This study
C314	I	?	1	C314-1	18	Clear	Circular, depressed, only external table affected	1.2	BFT	Yes			[19], This study
C318	MA	M	1	C318-1	19	Clear	Circular, depressed, only external table affected	0.9	BFT	Yes			This study
C326	ADOL	M?	3	C326-1	18	Clear	Oval, depressed, internal bevel, radiating fracture lines, adhered bone flakes	2.6	BFT	No		4r, 4s	This study
				C326-2	**37**, 18	Clear	Linear fracture, right, smooth	NA	BFT	No		4r	This study
				C326-3	**19**, 36	Clear	Oval, depressed, internal bevel	> 2.6	BFT	No			This study
C334	MA	M	1	C334-1	27	Clear	Linear fracture, oblique, smooth/jagged	NA	SFT	No		4t	This study
CAB	IA	?	1	CAB-1	**18**, 17	Probable	Oval, depressed, internal bevel, adhered bone flakes	> 3.3	BFT	No			This study
SJAPL.Trepa3	IA	?	1	SJAPL.Trepa3-1	35?	Clear	Trepanation by incision, both tables affected	> 2.3	TRE	No			[19], This study
SJAPL.01	ADOL	F?	1	SJAPL.01-1	**14**, 18	Clear	Trepanation by scraping, both tables affected, partially healed	> 6	TRE	Yes			[19], This study
SJAPL.03	IA	?	1	SJAPL.03-1	17	Clear	Oval, depressed, only external table affected	2.0	BFT	Yes			[19], This study

SJAPL.271	IA	?	1	SJAPL.271-1	18	Clear	Bump, circular	2.1	BFT	Yes			This study
SJAPL.387	ADOL	?	1	SJAPL.387-1	**16**, 9, 15	Clear	Linear fracture, oblique, smooth	NA	BFT	No			This study
SJAPL.Z	IA	?	1	SJAPL.Z-1	15	Clear	Circular, depressed, only external table affected	1.0	BFT	Yes			[19], This study
SJAPL.Z(b)	MA	M	2	SJAPL.Z(b)-1	**15**, 19, 18	Clear	Circular, punctuated, depressed, only external table affected	0.2	BFT	Yes			[19], This study
				SJAPL.Z(b)-2	18	Clear	Circular, punctuated, depressed, only external table affected	0.2	BFT	Yes			[19], This study

[1] Where: *I* = infant (0-6 years of age); *C* = child (7-11); *ADOL* = adolescent (12-19); *YA* = young adult (20-39); *MA* = middle adult (40-59); *OA* = old adult (>60); *IA* = indeterminate adult (>20).
[2] Where: *M* = male; *M?* = probable male; *F* = female; *F?* = probable female; *?* = ambiguous/indeterminate.
[3] Following the zonation method of Hussain et al. [32]. Zone numbers in bold indicate the zone in which the largest proportion of the lesion is located.
[4] Where: *BFT* = blunt-force trauma; *SFT* = sharp-force trauma; *PEN* = penetrative trauma; *TRE* = trepanation.
[5] Temporal sequence of blows following Puppe's rule [33], where identifiable.

Table S3. Postcranial injuries identified in SJAPL in this study, by injury type, healing and bones affected.

Bone affected	Injury type[1]								*TOTAL*
	Blunt-force		Sharp-force		Penetrative		Other		
	Unhealed	Healed	Unhealed	Healed	Unhealed	Healed	Unhealed	Healed	
Vertebrae					2	1			*3*
Ribs		3			1	2			*6*
Right scapula								1	*1*
Left scapula					1				*1*
Right clavicle		1							*1*
Left clavicle		1	1						*2*
Right humerus									
Left humerus			1						*1*
Right ulna		2				1[3]			*3*[3]
Left ulna		3[2]							*2*[2]

Right radius	2	2				1[3]			*4*[3]
Left radius		2[2]							*2*[2]
Right hand	1	1							*2*
Left hand									
Right coxal bone	1					1			*2*
Left coxal bone	1				1	1			*3*
Right femur	1	1							*2*
Left femur	3	1			1				*5*
Right tibia						1			*3*
Left tibia		2							
Right foot		3							*3*
Left foot		1							*1*
TOTAL	*9*	*22*	*2*		*6*	*7*		*1*	*47*

[1] Classified following M. Smith 's proposal [29].
[2] Includes a case affecting both the left ulna and radius of the same individual. Duplicate is discounted from totals where appropriate.
[3] Includes a case affecting both the right ulna and radius of the same individual. Duplicate is discounted from totals where appropriate

Table S4. Postcranial injuries identified at SJAP in this study, by individual.

ID	Cranium/ individual	Age[1]	Sex[2]	No. injuries	Location	Probability	Fracture description	Healing	Figure	Source
SJAPL.36(a)	–	IA	M	1	Right coxal bone	Clear	Embedded arrowhead	Yes		[19], This study
SJAPL.36(b)	–	IA	?	1	Left femur	Clear	V-shaped, oblique, smooth	No	5a	This study
SJAPL.43	–	IA	F	1	Left radius	Clear	Fracture callus, close to the wrist (Colles' fracture)	Yes		[19], This study
SJAPL.99	C161	YA	M	1	1st lumbar vertebra	Clear	Embedded arrowhead	No		[19], This study
SJAPL.154	C225	MA	M	1	Left coxal bone	Clear	Embedded arrowhead	No		[19], This study

SJAPL.158	–	IA	?	1	Right toe proximal phalanx	Clear	Fracture callus in the diaphysis	Yes		[19], This study
SJAPL.161	–	IA	?	1	Left femur	Clear	Oval, depressed	No	5b	This study
SJAPL.170	C198	ADOL	M	1	Right hemithorax	Clear	Embedded arrowhead	No		[19], This study
SJAPL.185	C215	MA	M	1	Right radius	Clear	Fracture callus, close to tuberosity (Galeazzi fracture)	Yes		[19], This study
SJAPL.199	C218	ADOL	M	1	Left scapula	Clear	Embedded arrowhead	No		[19], This study
SJAPL.210	–	IA	?	1	Left humerus	Probable	Linear, depressed	No		This study
SJAPL.217	–	YA	M	1	Left coxal bone	Clear	Transverse, right, jagged	No	5c	This study
SJAPL.325	C225	MA	M	1	Left rib fragment	Clear	Oval, depressed, arrowhead wound	Yes		[19], This study
SJAPL.326(a)	C221	ADOL	M	1	Left coxal bone	Clear	Embedded arrowhead	Yes		[19], This study
SJAPL.326(b)	C221	ADOL	M	1	Right scapula	Clear	Shoulder luxation	Yes		[19], This study
SJAPL.343	C227	YA	M	1	8th thoracic vertebra	Clear	Embedded arrowhead	No		[19], This study
SJAPL.343(b)	C227	YA	M	1	Right femur	Clear	Fracture callus in the diaphysis	Yes		This study
SJAPL.347	C224	ADOL	F	1	Proximal foot phalanx	Clear	Fracture callus in the diaphysis	Yes		[19], This study
SJAPL.357	C252	ADOL	F	1	Rib fragment	Clear	Fracture callus in the shaft	Yes		[19], This study
SJAPL.377	C234	YA	M	1	Right 3rd metacarpal bone	Clear	V-shaped, oblique, smooth	No	5d	This study
SJAPL.408	–	IA	?	1	Right coxal bone	Clear	V-shaped, oblique, smooth	No	5e	This study
SJAPL.465	–	IA	?	1	Right 5th metacarpal bone	Clear	Fracture callus in the diaphysis	Yes		[19], This study
SJAPL.559	C270	MA	M	1	Left clavicle	Clear	Fracture callus in the diaphysis	Yes		[19], This study
SJAPL.582	–	IA	F?	1	Left clavicle	Probable	Transverse, right, sharp	No		This study
SJAPL.636	C002(B1)	ADOL	F?	1	Thoracic vertebra fragment	Clear	Embedded arrowhead	Yes		[19], This study
SJAPL.653	C296	YA	M	1	Left rib fragment	Clear	Fracture callus in the angle	Yes		[19], This study
SJAPL.689	–	IA	?	1	Left ulna and radius	Clear	Fracture callus in the diaphysis (paired rotational fracture)	Yes		[19], This study
SJAPL.786	C212	YA	M	1	Right ulna and radius	Clear	Embedded arrowhead	Yes		[19], This study

SJAPL.871	–	ADOL	M	1	Right tibia	Clear	Oval, depressed, osteomyelitic changes, arrowhead wound	Yes		[19], This study
SJAPL.1985	–	IA	?	1	Left femur	Clear	Fracture callus in the diaphysis	Yes		[19], This study
SJAPL.1990	–	IA	?	1	Left ulna	Clear	Fracture callus in the diaphysis (tentatively diagnosed as parry fracture, in the absence of the ipsilateral radius)	Yes		[19], This study
SJAPL.A1	–	IA	?	1	Right toe proximal phalanx	Clear	Fracture callus in the diaphysis	Yes		[19], This study
SJAPL.A1.35	–	IA	?	1	Right radius	Clear	V-shaped, oblique/spiral, smooth	No	5f	This study
SJAPL.AB	–	IA	?	1	Right clavicle	Clear	Fracture callus in the diaphysis	Yes		[19], This study
SJAPL.B1(a)	–	IA	?	1	Left tibia	Clear	Fracture callus in the diaphysis	Yes		[19], This study
SJAPL.B1(b)	–	IA	?	1	Right radius	Clear	Longitudinal/curved, oblique, smooth	No	5g	This study
SJAPL.Z.2-4	–	IA	?	1	Rib fragment	Clear	Fracture callus in the angle	Yes		[19], This study
SJAPL.Z(a)	–	IA	?	1	Rib fragment	Probable	Oval, depressed, possible arrowhead wound	Yes		[19], This study
SJAPL.Z(b)	–	IA	?	1	3rd metatarsal bone fragment	Clear	Fracture callus in the diaphysis	Yes		[19], This study
SJAPL.Z(c)	–	IA	?	1	Left tibia	Clear	Fracture callus in the diaphysis	Yes		[19], This study
SJAPL.Z(d)	–	IA	?	1	Right radius	Clear	Fracture callus, close to the wrist (Colles' fracture)	Yes		[19], This study
SJAPL.SIN_SIGLA(a)	–	IA	?	1	Right ulna	Clear	Fracture callus in the diaphysis (tentatively diagnosed as parry fracture, in the absence of the ipsilateral radius)	Yes		[19], This study
SJAPL.SIN_SIGLA(b)	–	IA	?	1	Right ulna	Clear	Fracture callus in the diaphysis (tentatively diagnosed as parry fracture, in the absence of the ipsilateral radius)	Yes		[19], This study
SJAPL.SIN_SIGLA(c)	–	IA	?	1	Left ulna	Clear	Fracture callus in the diaphysis (tentatively diagnosed as parry fracture, in the absence of the ipsilateral radius)	Yes		[19], This study
SJAPL.SIN_SIGLA(d)	–	IA	?	1	Left femur	Clear	V-shaped, oblique, smooth	No		This study

SJAPL.SIN_SIGLA(e)	–	IA	?	1	Left femur	Clear	V-shaped, right, smooth	No	5h	This study
SJAPL.SIN_SIGLA(f)	–	IA	?	1	Femur indet.	Probable	V-shaped, mixed, jagged	No		This study

[1] Where: *ADOL* = adolescent (12-19); *YA* = young adult (20-39); *MA* = middle adult (40-59); *IA* = indeterminate adult (>20).

[2] Where: *M* = male; *M?* = probable male; *F* = female; *F?* = probable female; *?* = ambiguous/indeterminate.
